# PUF-8, a *C. elegans* ortholog of the RNA-binding proteins PUM1 and PUM2, is required for robustness of the cell death fate

**DOI:** 10.1242/dev.201167

**Published:** 2023-10-06

**Authors:** Jimei Xu, Yanwen Jiang, Ryan Sherrard, Kyoko Ikegami, Barbara Conradt

**Affiliations:** ^1^ Faculty of Biology, Center for Integrative Protein Sciences Munich (CIPSM), Ludwig-Maximilians-University, Munich, 82152 Planegg-Martinsried, Germany; ^2^ Department of Cell and Developmental Biology, Division of Biosciences, University College London, London WC1E 6BT, UK

**Keywords:** *C. elegans*, Apoptosis, CED-3 caspase, CED-9 BCL-2, Cell number homeostasis, Developmental robustness

## Abstract

During *C. elegans* development, 1090 somatic cells are generated, of which 959 survive and 131 die, many through apoptosis. We present evidence that PUF-8, a *C. elegans* ortholog of the mammalian RNA-binding proteins PUM1 and PUM2, is required for the robustness of this ‘survival and death’ pattern. We found that PUF-8 prevents the inappropriate death of cells that normally survive, and we present evidence that this anti-apoptotic activity of PUF-8 is dependent on the ability of PUF-8 to interact with *ced-3* (a *C. elegans* ortholog of caspase) mRNA, thereby repressing the activity of the pro-apoptotic *ced-3* gene. PUF-8 also promotes the death of cells that are programmed to die, and we propose that this pro-apoptotic activity of PUF-8 may depend on the ability of PUF-8 to repress the expression of the anti-apoptotic *ced-9* gene (a *C. elegans* ortholog of *Bcl2*). Our results suggest that stochastic differences in the expression of genes within the apoptosis pathway can disrupt the highly reproducible and robust survival and death pattern during *C. elegans* development, and that PUF-8 acts at the post-transcriptional level to level out these differences, thereby ensuring proper cell number homeostasis.

## Teaser

**Summary:** The RNA-binding protein PUF-8 is required for the highly reproducible and robust pattern of cell survival and cell death during *C. elegans* development.

## INTRODUCTION

The development of the nematode *Caenorhabditis elegans* is highly reproducible. Despite many sources of variation caused by intrinsic and extrinsic factors, *C. elegans* development culminates each time with the formation of an adult animal composed of the same set of 959 somatic cells (Sulston and Horvitz, 1977; Sulston et al., 1983). Therefore, *C. elegans* development exhibits extraordinary robustness and represents an ideal model for identifying robustness mechanisms that ensure normal development in the face of stochastic cell-to-cell differences in gene expression (Félix and Barkoulas, 2015; Maduro, 2015). The ‘cell death fate’ exemplifies developmental robustness: among the 1090 somatic cells generated during *C. elegans* development, the exact same 131 cells reproducibly undergo programmed cell death (Sulston and Horvitz, 1977; Sulston et al., 1983). This predictability is ideal for the analysis of the cell death fate and its robustness at single cell resolution. As a result of numerous studies, we have a molecular understanding of the pathway required for the execution of the cell death fate during *C. elegans* development, as well as the mechanisms through which the activity of this pathway is controlled (Horvitz, 2003; Lettre and Hengartner, 2006; Conradt et al., 2016). This makes the *C. elegans* cell death fate an ideal model for identifying robustness mechanisms.

Many of the 131 cells that are ‘programmed to die’ during *C. elegans* development die through apoptosis; their deaths are dependent on the conserved apoptosis pathway. This pathway is activated in cells programmed to die by the increase in gene expression above a critical ‘lethal’ threshold of the pro-apoptotic BH3-only gene *egl-1*. Once synthesized, EGL-1 protein directly interacts with the anti-apoptotic protein CED-9 (BCL-2) and this leads to CED-4 (Apaf1)-dependent apoptosome assembly, CED-3 (caspase) activation and CED-3-dependent apoptotic cell death (Horvitz, 2003; Lettre and Hengartner, 2006; Conradt et al., 2016). *egl-1* expression is controlled at the transcriptional and post-transcriptional level. At the level of transcription, *egl-1* expression is controlled by ‘lineage-specific’ transcription factors, each acting through a particular *cis*-acting element in the *egl-1* locus and mediating transcriptional activation of *egl-1* in one or a small number of ‘cell death’ lineages, i.e. lineages in which a cell death occurs (Conradt and Horvitz, 1999; Thellmann et al., 2003; Nehme and Conradt, 2008). The loss of such a lineage-specific transcription factor or the loss of its *cis*-acting element in the *egl-1* locus results in the loss of *egl-1* transcriptional activation in the respective cell death lineage(s) and the inappropriate survival of the cell(s) programmed to die. Both perturbations result in highly penetrant phenotypes, underscoring the importance of transcriptional control in the regulation of *egl-1* expression. At the post-transcriptional level, *egl-1* expression is controlled by members of the miR-35 and miR-58 families of microRNAs, which act through the 3′ UTR of *egl-1* mRNA to repress *egl-1* expression (Sherrard et al., 2017). This ensures that *egl-1* expression in cell death lineages reaches the critical lethal threshold only in the cell programmed to die. In mutants lacking miR-35 and miR-58 microRNAs, *egl-1* mRNA copy number is increased in mother cells, and this can lead to their inappropriate ‘precocious’ deaths. The penetrance of the ‘precocious death’ phenotype observed in animals lacking miR-35 microRNAs varies according to which of the three embryonic ‘waves of cell death’ is considered, ranging from 0% (1st wave) to 1.8% (2nd wave) and 8% (3rd wave). Therefore, in contrast to the loss of *egl-1* transcriptional control, the loss of microRNA-mediated post-transcriptional control of *egl-1* expression causes a weakly penetrant phenotype.

Gene expression is considered ‘noisy’, and stochastic differences in gene expression are thought to be a major target of robustness mechanisms (Félix and Barkoulas, 2015; Maduro, 2015). Indeed, the post-transcriptional control of *egl-1* expression through microRNAs can be considered a robustness mechanism: the involvement of a different transcription factor in essentially every cell death lineage causes differences – between cell death lineages – in the timing and level of *egl-1* transcriptional activation; through their repression of *egl-1* expression, miR-35 and miR-58 microRNAs act to level out these differences (Sherrard et al., 2017). Like microRNAs, RNA-binding proteins (RBPs) are crucial regulators of gene expression that exert their control at the post-transcriptional level by acting primarily through the 3′ UTR of mRNAs (Corley et al., 2020). Interestingly, compared with other factors involved in the control of gene expression (such as, for example, transcription factors), RBPs are found at relatively stable levels within cells in various organisms (Mittal et al., 2009; Joshi et al., 2012). For this reason, it has been proposed that RBPs may contribute to developmental robustness by levelling out cell-to-cell differences in gene expression (Mittal et al., 2009; Joshi et al., 2012). However, experimental evidence in support of this model remains elusive.

Here, we present evidence that PUF-8, a *C. elegans* ortholog of the mammalian RBPs PUM1 and PUM2, is required for the robustness of the cell death fate. We found that the loss of *puf-8* can cause cells that normally survive to die inappropriately. Surprisingly, we found that the loss of *puf-8* can also cause cells that are programmed to die to survive inappropriately. Our results furthermore indicate that rather than targeting the expression of *egl-1*, the PUF-8 protein targets the expression of genes encoding other components of the apoptosis pathway, i.e. *ced-9*, *ced-4* and *ced-3*. To our knowledge, at least, this is the first report implicating a member of the PUF family of RBPs in developmental robustness.

## RESULTS

### In embryonic cells that normally do not die *puf-8* has anti-apoptotic activity

**Fig. 1.**
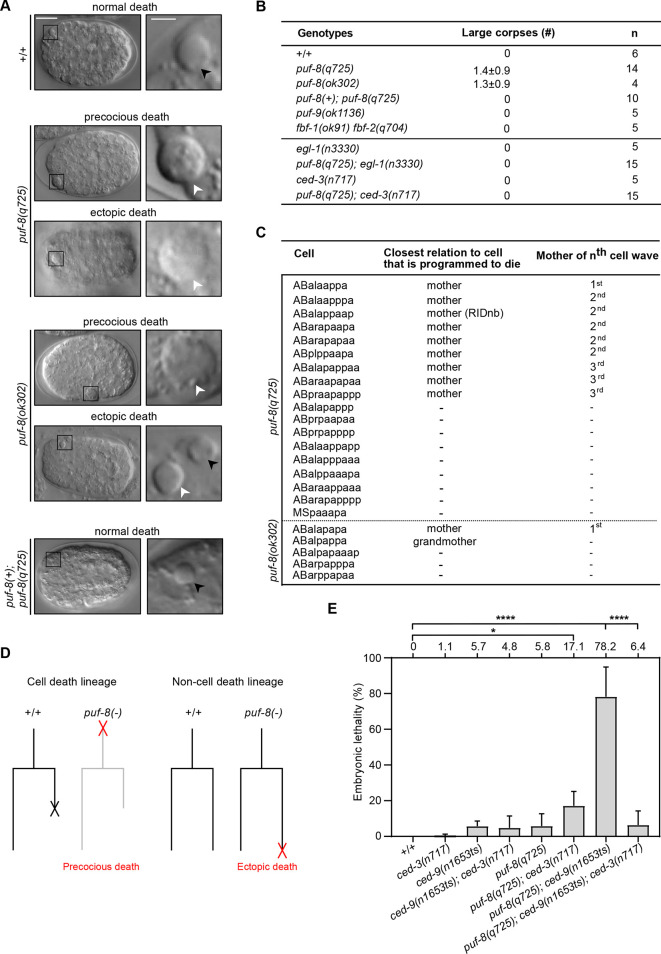
***puf-8* has anti-apoptotic activity.** (A) DIC images of cell corpses in embryos of genotypes indicated. Abnormally large corpses are present in *puf-8(q725)* and *puf-8(ok302)* embryos (white arrowheads). Normal corpses are indicated by black arrowheads. Scale bars: 10 μm for embryos; 2 μm for individual corpses. (B) Average numbers of large corpses per embryo during embryogenesis before ventral enclosure in different genotypes. +/+ indicates wild type. The number of embryos examined (*n*) is indicated. (C) Identities of large corpses in *puf-8(q725)* and *puf-8(ok302)* embryos determined by cell lineage analysis. Only direct relationships to a cell death (i.e. mother or grandmother) were considered. (D) Schematics of defects detected in *puf-8* mutants in cell death lineage (precocious death) and in non-cell death lineage (ectopic death). (E) Percent embryonic lethality in different genotypes. The number of embryos analyzed (*n*) is 2940 for +/+, 2411 for *ced-3(n717)*, 785 for *ced-9(n1653ts)*, 3307 for *puf-8(q725)*, 2947 for *ced-9(n1653ts); ced-3(n717)*, 415 for *puf-8(q725); ced-9(n1653ts)*, 3272 for *puf-8(q725); ced-3(n717)* and 1457 for *puf-8(q725); ced-9(n1653ts); ced-3(n717)*. The average embryonic lethality of each genotype is given at the top. Data are mean±s.d. *P≤0.05; ****P≤0.0001 (two-tailed Mann–Whitney test).

The gene *puf-8* encodes one of 11 members of the PUF [PUMILIO and FBF (*fem-3* mRNA binding factor)] family of RNA-binding proteins (RBPs) in *C. elegans* and is most similar to the genes encoding mammalian PUM1 and PUM2 (Wickens et al., 2002; Wang et al., 2018). *puf-8* has been shown to function in the hermaphrodite germline and in somatic tissues of developing larvae and adults (Walser et al., 2006; Arey et al., 2019; D'Amico et al., 2019; Wang and Voronina, 2020). As part of a small scale screen for RBP genes with a role in developmental robustness, using a four-dimensional (4D) microscope system (Schnabel et al., 1997), we followed embryonic development of embryos homozygous for *q725* or *ok302*: two strong loss-of-function mutations of *puf-8* (Subramaniam and Seydoux, 2003; Bachorik and Kimble, 2005). Both mutations are deletions and result in the synthesis of mutant PUF-8 proteins that lack seven or eight of the eight Puf repeats, and thus do not have a functional Pumilio homology domain (Pum-HD) (Fig. S1A). Most cells that die during *C. elegans* development turn into refractile ‘corpses’ ∼2.5 μm in diameter within 20-30 min of being generated (Fig. 1A; +/+, black arrowheads). Apart from these ‘normal’ corpses, in *puf-8* mutant embryos, we also detected corpses larger than 2.5 μm in diameter [Fig. 1A; *puf-8*(*q725*) or *puf-8*(*ok302*), white arrowheads]. We quantified the number of these ‘large’ corpses until the completion of ventral closure (280 min or 330 min after the first cleavage of the zygote at 25°C in wild-type and *puf-8* mutants, respectively) and found an average of 1.4 or 1.3 large corpses per embryo in *puf-8*(*q725*) or *puf-8*(*ok302*) mutants, respectively (Fig. 1B). A *puf-8*(+) transgene (Fig. S1B, P*_puf-8_puf-8*) rescues this ‘large corpses’ phenotype in *puf-8*(*q725*) mutants [Fig. 1A,B; *puf-8*(*+*); *puf-8*(*q725*)]. *puf-8* is part of operon CEOP2292 on chromosome II, which also includes the gene *C30G12.6* (Fig. S1A). (*C30G12.6* encodes a protein of unknown function.) To rule out the possibility that the large corpses phenotype observed in *puf-8*(*q725*) and *puf-8*(*ok302*) animals is the result of the loss of *C30G12.6*, we analysed animals homozygous for the deletion *ok2398*, which removes most of the coding region of *C30G12.6* (Fig. S1A). We failed to detect large corpses in *C30G12.6*(*ok2398*) animals (Fig. S2A). Furthermore, a *C30G12.6* (+) transgene (Fig. S1C, P_*puf-8*_*C30G12.6*) failed to rescue the large corpses phenotype in *puf-8*(*q725*) mutants [Fig. S2A; *C30G12.6*(*+*); *puf-8*(*q725*)]. We conclude that the loss of *puf-8*, but not *C30G12.6*, causes a large corpses phenotype.

Using cell lineage analyses, we determined the identities of the large corpses in *puf-8* mutants and found that all are cells that normally survive (Fig. 1C). Among 18 large corpses in *puf-8*(*q725*) mutants, nine were identified as mothers of a cell death and nine as cells from non-cell death lineages (i.e. cell lineages in which normally no cell death occurs). The mothers identified are mothers of 1st, 2nd and 3rd wave cell deaths. Among five large corpses in *puf-8*(*ok302*) mutants, one was identified as a mother of a cell death, one as a grandmother of a cell death and three as cells from non-cell death lineages (Fig. 1C). Hence, the loss of *puf-8* causes both precocious and ‘ectopic’ cell death [Fig. 1D; *puf-8*(*-*)]. Based on the number of cells present at the completion of ventral closure (∼400 cells/embryo, of which ∼100 are ancestors of cells fated to die), the penetrance of the precocious or ectopic cell death phenotypes in *puf-8* mutants is ∼0.7% or ∼0.2%, respectively. To determine whether the precocious and ectopic deaths observed are dependent on the apoptosis pathway, we tested whether a loss-of-function mutation of *egl-1*(*n3330*) or *ced-3*(*n717*) suppresses the large corpses phenotype in *puf-8*(*q725*) mutants. *egl-1*(*n3330*) and *ced-3*(*n717*) essentially block all apoptotic cell deaths that occur during development (Ellis and Horvitz, 1986; Sherrard et al., 2017) (Fig. 1B). We found that in both double mutants, normal corpses and large corpses are no longer present [Fig. 1B; *puf-8*(*q725*); *egl-1*(*n3330*), *puf-8*(*q725*); *ced-3*(*n717*)]. Therefore, the presence of large corpses in *puf-8* mutants is the result of the precocious or ectopic activation of the apoptosis pathway.

The loss of the anti-apoptotic gene *ced-9* causes precocious and ectopic cell death, resulting in embryonic lethality referred to as ‘Emb’ phenotype (Hengartner et al., 1992). To determine whether the loss of *puf-8* enhances the loss of *ced-9*, we took advantage of the weak temperature-sensitive (ts) *ced-9* loss-of-function mutation *n1653*ts (Hengartner et al., 1992). We found that at the semi-permissive temperature of 20°C, there are low levels of embryonic lethality in *ced-9*(*n1653*ts) and *puf-8*(*q725*) single mutants (5.7% and 5.8%, respectively); however, in *puf-8*(*q725*)*; ced-9*(*n1653*ts) double mutants, embryonic lethality increases to 78.2% [Fig. 1E; *puf-8*(*q725*)*; ced-9*(*n1653*ts)]. Furthermore, we found that the loss of *ced-3* suppresses the embryonic lethality observed in *puf-8*(*q725*)*; ced-9*(*n1653*ts) double mutants (6.4%) [Fig. 1E; *puf-8*(*q725*)*; ced-9*(*n1653*ts); *ced-3*(*n717*)]. Embryonic lethality in *puf-8*(*q725*)*; ced-9*(*n1653*ts) animals is therefore caused by the inappropriate activation of the apoptosis pathway. Based on these observations, we conclude that in embryonic cells that normally do not die, the loss of *puf-8* can lead to their inappropriate death. Therefore, *puf-8* has anti-apoptotic activity that ensures the survival of these cells.

*puf-8* is most similar to the gene *puf-9*, which also encodes an ortholog of human PUM1 and PUM2 (Wickens et al., 2002). Indeed, PUF-8 and PUF-9 proteins are more similar to human PUM1 and PUM2 than to any other member of the *C. elegans* family of PUF proteins. [The PUM1/2 and PUF-8/9 duplication is likely to have occurred before the separation of nematodes and vertebrates (Spassov and Jurecic, 2003).] To determine whether the large corpses phenotype observed is specific to the loss of *puf-8*, we analysed animals lacking a functional *puf-9* gene, *puf-9*(*ok1136*) (Nolde et al., 2007), but failed to detect large corpses [Fig. 1B; *puf-9*(*ok1136*)]. The genes *fbf-1* and *fbf-2* also encode members of the *C. elegans* family of PUF proteins and are functionally redundant (Wickens et al., 2002; Walser et al., 2006). For this reason, we analyzed animals lacking both genes [*fbf-1*(*q91*) *fbf-2*(*ok704*)] (Crittenden et al., 2002). We detected no large corpses in the double mutant [Fig. 1B; *fbf-1*(*q91*) *fbf-2*(*ok704*)]. Therefore, the loss of PUF family RBPs does not generally result in precocious or ectopic cell death, and, hence, the anti-apoptotic activity observed in cells fated to survive may be specific to PUF-8.

### In embryonic cells that are programmed to die, *puf-8* has pro-apoptotic activity

**Fig. 2.**
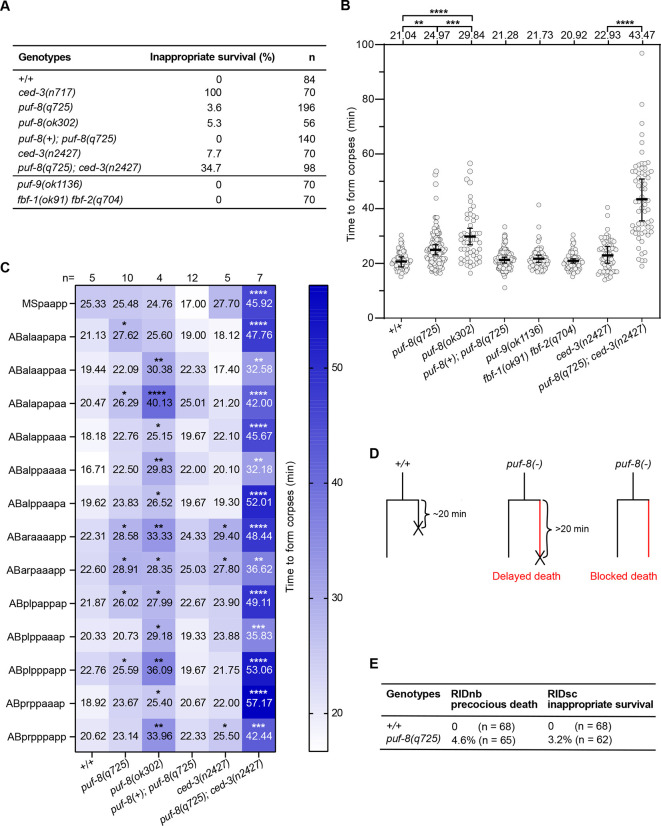
***puf-8* has pro-apoptotic activity.** (A) Percent inappropriate survival during the 1st wave of cell death (first 14 cell deaths during embryogenesis) in different genotypes. +/+ indicates wild type. The number of cells examined (*n*) is indicated. (B) The time to form a corpse was measured in minutes for cell deaths of the 1st wave in the genotypes indicated. Grey dots represent the values for individual cell deaths. The number of cell deaths analyzed (*n*) is 70 for +/+, 53 for *puf-8*(*q725*), 135 for *puf-8*(*ok302*), 126 for *puf-8*(+); *puf-8*(*q725*), 70 for *puf-9*(*ok1136*), 70 for *fbf-1*(*ok91*) *fbf-2*(*q704*), 67 for *ced-3*(*n2427*) and 65 for *puf-8*(*q725*); *ced-3*(*n2427*). Average values are given above each dataset. Data are mean±s.d. **P≤0.01; ***P≤0.001; ****P≤0.0001 (two-tailed Mann–Whitney test). (C) The time taken to form corpses in individual cell death lineages of the 1st wave of cell death in various genotypes. The average time to form corpses is given for each genotype. Values are coloured according to the scale on the right. The number of embryos (*n*) examined for each genotype is given at the top. *P≤0.05; **P≤0.01; ***P≤0.001; ****P≤0.0001 by two-way ANOVA with multiple comparison to wild type. (D) Schematics of defects detected in *puf-8* mutants in cell death lineages (delayed death and blocked death). (E) Percentage of precocious RIDnb death and inappropriate RIDsc survival in wild type (+/+) and *puf-8(q725)* mutant. The RID lineage was identified in embryos using the reporter *P_unc-3_unc-3::gfp* (*xdEx1091*). The number of +/+ and *puf-8(q725)* mutant embryos examined (*n*) are 68 and 65 for the RIDnb death assay, and 68 and 62 for the RIDsc survival assay, respectively. The data are presented as percentages.

During our cell lineage analyses, we noticed that in *puf-8* mutants, some cells that normally die, inappropriately survive. To determine whether PUF-8 also plays a role in cells fated to die, we followed the fate of the 14 cells that die during the 1st wave of embryonic cell death (140-200 min or 150-210 min after the first division of the zygote at 25°C in wild-type or *puf-8* mutant embryos, respectively) (Sulston et al., 1983). In wild-type embryos, all 14 cells reproducibly die, resulting in 0% inappropriate survival (Fig. 2A). In contrast, in *puf-8*(*q725*) or *puf-8*(*ok302*) mutants, some of these cells fail to die, resulting in 3.6% and 5.3% inappropriate survival, respectively, and hence a cell-death abnormal or Ced phenotype. For comparison, the strong *ced-3* loss-of-function mutation *n717* causes 100% inappropriate survival and the weak *ced-3* loss-of-function mutation *n2427* causes 7.7% inappropriate survival (Ellis and Horvitz, 1986; Shaham et al., 1999) (Fig. 2A). Furthermore, we found that the cells that die in *puf-8* mutants, die more slowly. Whereas in wild-type embryos, 1st wave cell deaths turn into refractile corpses in a mean time of 21.04 min, it takes them 24.97 min and 29.84 min in *puf-8*(*q725*) or *puf-8*(*ok302*) mutants, respectively (Fig. 2B). [Of note, the difference between *puf-8*(*q725*) and *puf-8*(*ok302*) is statistically significant and may be caused by a mutation in the *puf-8*(*ok302*) background.] The effect observed is cell lineage specific: the cell death in the MS lineage (MSpaapp) is not affected; the cell deaths in the AB lineage are all affected but to varying degrees (Fig. 2C). The Ced phenotype and delayed cell death observed in *puf-8*(*q725*) animals is rescued by the *puf-8*(+) transgene [Fig. 2A-C; *puf-8*(*+*)*; puf-8*(*q725*)]. To investigate whether these defects could be a consequence of the loss of *C30G12.6*, we analyzed *C30G12.6(ok2389)* animals. We failed to detect inappropriately surviving cells and we also failed to detect a significant increase in the time it took cells to die (Fig. S2B). In addition, a *C30G12.6*(+) transgene failed to rescue either the Ced phenotype or the delay in cell death observed in *puf-8*(*q725*) animals (Fig. S2B). Therefore, we conclude that the loss of *puf-8* but not *C30G12.6* causes a Ced phenotype and a delay in cell death.

Next, we tested whether the loss of *puf-8* enhances the phenotype caused by the weak *ced-3* loss-of-function mutation *n2427* (Shaham et al., 1999). We found that *puf-8*(*q725*) increases inappropriate survival in *ced-3*(*n2427*) animals from 7.7% to 34.7%. In addition, *puf-8*(*q725*) increases the time taken for cells to die in *ced-3*(*n2427*) animals from 22.93 min to 43.47 min [Fig. 2A-C; *puf-8*(*q725*); *ced-3*(*n2427*)]. Together, these results demonstrate that the loss of *puf-8* compromises apoptotic cell death, leading to delayed or blocked cell death (Fig. 2D). Therefore, in cells that are programmed to die, *puf-8* has pro-apoptotic activity. This pro-apoptotic activity ensures that these cells adopt the cell death fate all the time and that the cell death fate is swiftly executed.

Finally, to determine whether the Ced phenotype and the delay in cell death observed is specific to the loss of *puf-8*, we analyzed *puf-9*(*ok1136*) and *fbf-1*(*q91*) *fbf-2*(*ok704*) animals. We did not observe inappropriate survival or delayed cell death in these mutants (Fig. 2A,B). Therefore, the loss of PUF family RNA-binding proteins does not generally result in a Ced phenotype or in delayed cell death, and the pro-apoptotic activity of *puf-8* in cells fated to die may be specific to *puf-8*.

### The loss of *puf-8* impacts the copy numbers of mRNAs encoding components of the apoptosis pathway

PUF proteins bind to specific sequence motifs in the 3′ UTR of mRNAs and impact gene expression by affecting mRNA turnover and mRNA translation (Wickens et al., 2002; Goldstrohm et al., 2018). The motifs targeted by PUF-8 have previously been determined (Opperman et al., 2005). Using MEME (Multiple Em for Motif Elicitation) analysis (Bailey et al., 2009) and FIMO (Find Individual Motif Occurrences) (Grant et al., 2011), we took advantage of these motifs to identify potential ‘PUF binding elements’ (PBEs) in the 3′ UTRs of mRNAs that encode components of the apoptosis pathway (*egl-1, ced-9, ced-4* and *ced-3* mRNAs). Using this approach, we did not identify PBEs in the *egl-1* 3′ UTR. However, we identified five, two and six PBEs in the 3′ UTRs of *ced-9*, *ced-4* and *ced-3*, respectively, making them potential direct targets of PUF-8 (Fig. S3).

To determine whether *puf-8* impacts cell death by affecting turnover of mRNAs encoding components of the core apoptosis pathway, we used Single Molecule RNA Fluorescence *In Situ* Hybridization (smRNA FISH) to analyse the copy numbers of *egl-1*, *ced-9*, *ced-4* and *ced-3* mRNAs in a specific cell death lineage: the RID (Ring Interneuron D) lineage. The RID neuroblast (RIDnb) divides around 330 min after the first division of the zygote at 25°C to produce a daughter cell that survives and differentiates into the RID neuron and its sister cell (RIDsc), which is programmed to die during the 2nd wave of cell death (Sulston et al., 1983). Importantly, the RIDnb is one of nine mothers that we had identified as precociously dying in *puf-8*(*q725*) animals (Fig. 1C). An *unc-3* transgene (P*_unc-3_unc-3::gfp*) is expressed in the RIDnb and its daughter cells in embryos, and can be used to analyse survival and cell death in this lineage (Wang et al., 2015). To confirm that *puf-8* has anti-apoptotic and pro-apoptotic activities in the RID lineage, we analyzed wild-type and *puf-8*(*q725*) embryos carrying the *unc-3* transgene. We found that in wild-type embryos, 0% of the RIDnb precociously died, and 0% of the RIDsc inappropriately survived (Fig. 2E). In contrast, in *puf-8*(*q725*) embryos, 4.6% (3 out of 65) of the RIDnb precociously died and 3.2% (2 out of 62) of the RIDsc had not died at the end of our recordings (twofold stage) and therefore most probably inappropriately survived (Fig. 2E). These results confirm that *puf-8* has anti-apoptotic and pro-apoptotic activities in the RID lineage.

**Fig. 3.**
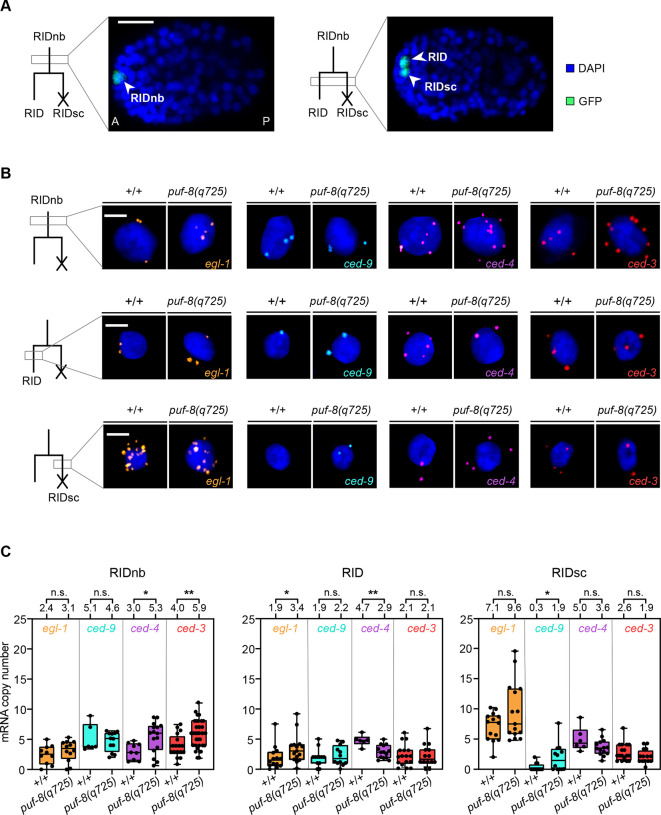
**Copy numbers of *egl-1*, *ced-9*, *ced-4* and *ced-3* mRNAs in the RID lineage.** (A) Fixed wild-type embryos with RIDnb (left) and its two daughter cells (right), RID and RIDsc, as identified by the reporter P*_unc-3_unc-3::gfp* (*xdEx1091*) and DAPI staining. The anterior-posterior axis (A↔P) is indicated. Scale bar: 10 μm. (B) smRNA FISH analysis in RIDnb (top row), RID (middle row) and RIDsc (bottom row). Confocal images of representative RIDnb, RID and RIDsc are shown for wild type (+/+) and *puf-8(q725)* as indicated. Nuclei are stained with DAPI. Scale bars: 2 μm. (C) Quantification of *egl-1*, *ced-9*, *ced-4* and *ced-3* mRNA copy numbers in RIDnb, RID and RIDsc in wild type (+/+) and *puf-8(q725)*. Each black dot represents the mRNA copy number in one individual cell. The numbers of RIDnb analyzed (*n*) for *egl-1*, *ced-9*, *ced-4* and *ced-3* mRNAs are 10, 6, 9 and 17 in wild type (+/+) and 12, 12, 16 and 34 in *puf-8(q725)*, respectively. The numbers of RID analyzed (*n*) for *egl-1*, *ced-9*, *ced-4* and *ced-3* mRNAs are 14, 12, 6 and 17 in wild type (+/+) and 15, 12, 14 and 14 in *puf-8(q725)*, respectively. The numbers of RIDsc analyzed (*n*) for *egl-1*, *ced-9*, *ced-4* and *ced-3* mRNAs are 14, 12, 6 and 17 in wild type (+/+) and 13, 12, 14 and 14 in *puf-8(q725)*, respectively. Average mRNA copy numbers are shown on top of the graphs. Boxes represent the interquartile range; whiskers represent the minimum and maximum. Data were plotted according to Tukey's test. *P≤0.05; **P≤0.01 (two-tailed Mann–Whitney test).

Next, we used the *unc-3* transgene to identify the RID lineage in fixed smRNA FISH-labelled embryos at the time the RIDnb divides (Fig. 3A). Copy numbers of mRNAs were quantified in individual GFP-positive cells from image stacks obtained by confocal microscopy. Briefly, the total smRNA FISH signal in an individual RIDnb, RID or RIDsc was quantified and divided by the signal of a single mRNA molecule to determine mRNA copy number in that cell (see Materials and Methods). We first analyzed the RIDnb in wild-type animals, observing mean copy numbers of 2.4 for *egl-1* mRNA, 5.1 for *ced-9* mRNA, 3.0 for *ced-4* mRNA and 4.0 for *ced-3* mRNA (Fig. 3B,C; RIDnb). Whereas the mean copy numbers of *egl-1* mRNA and *ced-9* mRNA are not significantly altered in *puf-8*(*q725*) mutants, the mean copy numbers of *ced-4* mRNA and *ced-3* mRNA are significantly increased to 5.3 (1.76-fold increase) and 5.9 (1.48-fold increase), respectively. Next, we determined mRNA copy numbers in the RID and RIDsc shortly after the division of the RIDnb. In the RID, we detected significant changes in the mean copy numbers of *egl-1* mRNA (a 1.8-fold increase) and *ced-4* mRNA (a ∼40% decrease) (Fig. 3B,C; RID). The only significant change we detected in the RIDsc is a ∼6.3-fold increase (from 0.3 to 1.9) in the mean copy number of *ced-9* mRNA (Fig. 3B,C; RIDsc). In summary, the loss of *puf-8* impacts the copy numbers of mRNAs encoding pro- and anti-apoptotic components in the RID lineage. Importantly, it significantly increases the copy numbers of pro-apoptotic *ced-4* and *ced-3* mRNAs in the RIDnb, a cell that normally does not die, and it significantly increases the copy number of anti-apoptotic *ced-9* mRNA in the RIDsc, a cell that normally dies.

### PUF-8 protein interacts physically with *ced-3* mRNA

**Fig. 4.**
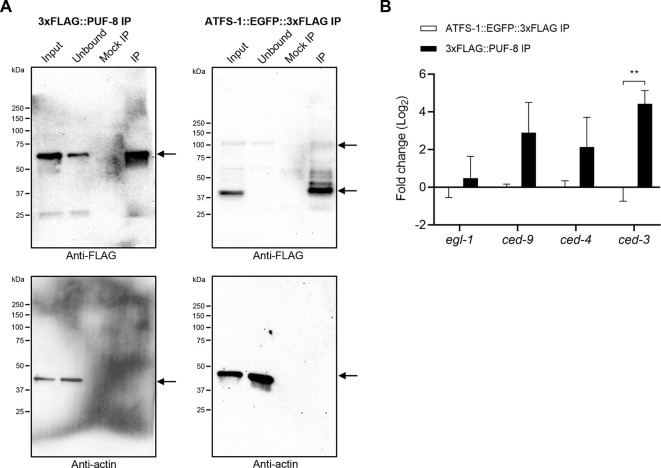
**PUF-8 protein interacts physically with *ced-3* mRNA.** (A) Western blots of 3xFLAG::PUF-8 and ATFS-1::EGFP::3xFLAG immunoprecipitations (IPs) probed with antibodies against FLAG or actin (control). Mouse anti-FLAG antibodies and mouse anti-actin antibodies were used at a 1:2000 dilution in blocking buffer. Goat anti-mouse IgG (light chain specific) antibodies were used at a 1:5000 dilution in the blocking buffer. (B) Real-time quantitative PCR (RT qPCR) of mRNAs in 3xFLAG::PUF-8 and ATFS-1::EGFP::3xFLAG IPs. Enrichment of mRNAs present in 3xFLAG::PUF-8 IP compared with ATFS-1::EGFP::3xFLAG IP is shown for four biological replicates. Data were normalized to endogenous control *tbg-1* mRNA and analyzed using the ΔΔCT method. Data are mean±s.e.m. and are analyzed using an unpaired two-tailed Student's *t*-test; **P<0.01.

PUF-8 protein impacts the expression of target genes primarily by directly binding to PBEs within the 3′ UTRs of target mRNAs (Mainpal et al., 2011; Vaid et al., 2013; Park et al., 2020). As mentioned above, we identified five, two or six PBEs in the 3′ UTRs of *ced-9*, *ced-4* or *ced-3*, respectively (Fig. S3). To determine whether PUF-8 protein physically interacts with *ced-9*, *ced-4* or *ced-3* mRNAs *in vivo*, we performed immunoprecipitations (IPs) using PUF-8 protein, tagged at its N terminus with three FLAG tags (3xFLAG::PUF-8) produced from the endogenous *puf-8* locus. Specifically, using a FLAG tag-specific antibody, we precipitated 3xFLAG::PUF-8 protein from *C. elegans* lysates generated from mixed-stage *C. elegans* cultures (Fig. 4A, 3xFLAG::PUF-8 IP). Using the same antibody, as a control, we precipitated the protein ATFS-1::EGFP::3xFLAG from mixed-stage lysates (Fig. 4A, ATFS-1::EGFP::3xFLAG IP) (where ATFS-1 is activating transcription factor associated with stress 1) (Haynes et al., 2010). Precipitated 3xFLAG::PUF-8 and ATFS-1::EGFP::3xFLAG proteins were then analyzed for co-precipitating *egl-1*, *ced-9*, *ced-4* or *ced-3* mRNAs using quantitative PCR (qPCR). We found that compared with the control protein ATFS-1::EGFP::3xFLAG, there is significant enrichment (more than fourfold) of *ced-3* mRNA in the 3xFLAG::PUF-8 precipitate (Fig. 4B). There is also a more than twofold enrichment of *ced-9* or *ced-4* mRNAs in the 3xFLAG::PUF-8 precipitate (2.9-fold for *ced-9* and 2.1-fold for *ced-4*); however, these are not statistically significant. In contrast and consistent with the finding that there are no PBEs in the 3′ UTR of *egl-1*, there is essentially no enrichment of *egl-1* mRNA (0.4-fold). Based on these findings, we conclude that PUF-8 protein interacts physically with *ced-3* mRNA *in vivo*, and that it may also interact physically with *ced-9* and *ced-4* mRNAs.

### The anti-apoptotic activity of PUF-8 protein is mediated by PUF-binding elements in the 3′ UTR of *ced-3* mRNA

**Fig. 5.**
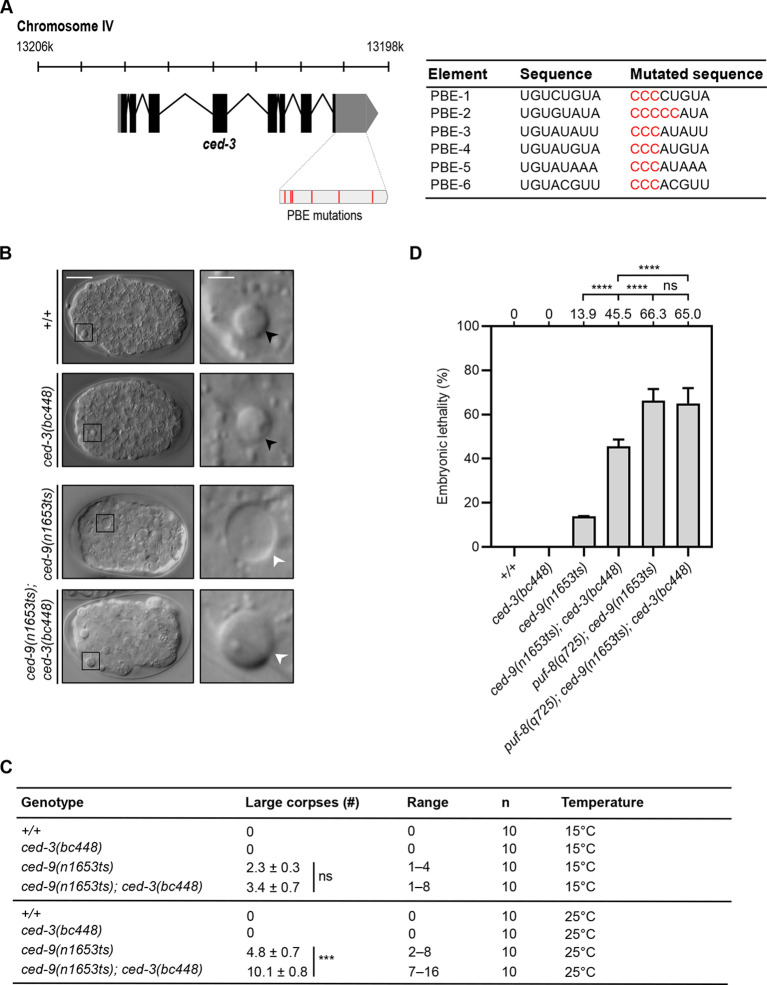
**PUF-8 binding elements (PBEs) in the *ced-3* 3′ UTR contribute to *puf-8*-dependent repression of *ced-3* expression.** (A) Schematic of endogenous *ced-3* locus on chromosome IV and *ced-3* mRNA, including 3′ UTR with PBE mutations introduced by CRISPR/Cas-mediated genome editing to generate *ced-3*(*bc448*) indicated in red (left). Sequence changes introduced into each of the six PBEs are indicated in red in the table (right). (B) DIC images of cell corpses in embryos of the genotypes indicated. The images of embryos are shown on the left with genotypes indicated and enlarged insets (5×) of cell corpses are shown on the right. Large corpses are indicated by white arrowheads; normal cell corpses are indicated by black arrowheads. Scale bars: 10 μm for embryo images; 2 μm for cell corpse images. (C) Average numbers of large corpses per embryo until ventral enclosure in different genotypes at 15°C and 25°C. The number of embryos analyzed (*n*) is 10 for each genotype and is indicated in the table. Data are mean±s.e.m., ns indicates no significance; ***P<0.001 (two-tailed unpaired Student's test). (D) Percent embryonic lethality in animals of different genotypes. Embryonic lethality assays were performed at 20°C. The sample sizes for wild type (*+/+*), *ced-3(bc448)*, *ced-9(n1653ts)*, *ced-9(n1653ts); ced-3(bc448)*, *puf-8(q725); ced-9(n1653ts)* and *puf-8(q725); ced-9(n1653ts); ced-3(bc448)* was 323, 343, 426, 412, 154 and 206, respectively. Data are mean±s.e.m., ns indicates no significance, ****P<0.0001 (two-tailed Fisher's exact test). (Of note, the percentage embryonic lethality observed for *puf-8(q725); ced-9(n1653ts)* in the experiment presented in Fig. 1E is 78.2%. In the experiment presented in this figure it is 66.3%. The two sets of experiments were performed by two different individuals in two different laboratories.)

*puf-8* has anti-apoptotic activity in cells that normally do not die (including the RIDnb), the copy number of *ced-3* mRNA is significantly increased in the RIDnb in embryos lacking *puf-8* function and PUF-8 protein physically interacts with *ced-3* mRNA *in vivo*. To determine whether the anti-apoptotic function of *puf-8* is mediated through the interaction of PUF-8 protein with PBEs found in the 3′ UTR of *ced-3* mRNA, we used CRISPR/Cas-mediated genome editing to mutate the six PBEs in the *ced-3* 3′ UTR (Fig. S3) and analyzed embryos homozygous for the resulting allele *ced-3*(*bc448*) (Fig. 5A). We found no large corpses indicative of precocious or ectopic cell death in *ced-3*(*bc448*) embryos grown at 15°C or 25°C (Fig. 5B,C). However, we found that in the background of the weak temperature-sensitive (ts) *ced-9* loss-of-function mutation *n1653*ts, *ced-3*(*bc448*) significantly enhanced the number of large corpses per embryo at 25°C (from 4.8 to 10.1) (Fig. 5B,C). In addition, we found that at 20°C, *ced-3*(*bc448*) significantly increased embryonic lethality in the *ced-9*(*n1653*ts) background from 13.9% to 45.5% (Fig. 5D). These observations demonstrate that in the *ced-9*(*n1653*ts) background, *ced-3*(*bc448*) promotes precocious and ectopic cell death, which suggests that *bc448* increases the activity of the endogenous *ced-3* gene and, hence, represents a weak gain-of-function allele of *ced-3*. To our knowledge, at least, this is the first *ced-3* allele reported that increases *ced-3* activity and enhances rather than suppresses the loss of *ced-9*. In contrast to *ced-9*(*n1653*ts); *ced-3*(*bc448*), which causes 45.5% lethality, *puf-8*(*q725*); *ced-9*(*n1653*ts) causes 66.3% lethality at 20°C (Fig. 5D). This suggests that in the context of the anti-apoptotic function of *puf-8*, PUF-8 protein has pro-apoptotic targets other than *ced-3* mRNA. (We speculate that this additional target could be *ced-4* mRNA.) Alternatively, there may be PBEs in the *ced-3* 3′ UTR in addition to the six that we identified and inactivated to which PUF-8 can bind and repress *ced-3* expression. Finally, and importantly, we found that *ced-3*(*bc448*) does not increase embryonic lethality in *puf-8*(*q725*); *ced-9*(*n1653*ts) animals (66.3% versus 65%; Fig. 5D). This indicates that the increase in embryonic lethality caused by *ced-3*(*bc448*) in the *ced-9*(*n1653*ts) background (from 13.9% to 45.5%) is the result of the loss of PUF-8 binding to *ced-3* mRNA (and, hence, the loss of PUF-8-dependent repression of *ced-3* expression) rather than the loss of binding of, for example, another member of the family of PUF RBPs to the six PBEs. In conclusion, these observations provide evidence that the anti-apoptotic activity of PUF-8 protein is mediated (at least in part) by PUF-binding elements in the 3′ UTR of *ced-3* mRNA.

## DISCUSSION

### *puf-8* fine-tunes apoptosis during *C. elegans* development

We present evidence that *puf-8* is required for the robustness of the highly reproducible pattern of cell survival and cell death during the development of somatic lineages in *C. elegans*. In *puf-8* mutants, some cells that would normally die, inappropriately survive. Furthermore, the loss of *puf-8* significantly enhances the cell-death abnormal (Ced) phenotype of animals homozygous for a weak *ced-3* loss-of-function mutation. On the other hand, we also find that some cells that would normally survive undergo inappropriate cell death in *puf-8* mutants. Furthermore, the loss of *puf-8* significantly enhances ectopic and precocious apoptotic death caused by the partial loss of *ced-9* function, resulting in embryonic lethality (Emb phenotype). By exhibiting both anti- and pro-apoptotic activities, the *puf-8* gene can be considered to have dual and opposing roles in the control of apoptosis. We propose that these dual and opposing roles ‘fine-tune’ apoptosis, thereby contributing to cell number homeostasis during the development of somatic tissues in *C. elegans*.

Interestingly, *puf-8* also has dual and opposing roles in a process relevant for cell number homeostasis in the hermaphrodite germline (Kimble and Crittenden, 2007; Wang and Voronina, 2020). *puf-8* promotes mitosis in germline stem cells (GSCs) and therefore GSC proliferation, and the loss of *puf-8* result in a reduction in the number of GSCs (Bachorik and Kimble, 2005; Ariz et al., 2009). However, *puf-8* also promotes the ability of GSCs to enter meiotic prophase and differentiate, and the loss of *puf-8* results in germline tumours most likely because GSCs fail to enter meiotic prophase and instead continue to proliferate mitotically (Racher and Hansen, 2012). Therefore, *puf-8* both promotes and inhibits GSC proliferation. In addition, like the *puf-8* phenotypes in the context of apoptosis, the *puf-8* phenotypes in the context of GSC proliferation are of low penetrance, and the loss of *puf-8* enhances the phenotypes caused by mutations of other genes that regulate GSC proliferation. For example, like the loss of *puf-8*, the loss of the gene *mex-3* (which encodes a KH domain-containing RNA-binding protein) results in a mild reduction in the number of GSCs. However, in animals lacking both *puf-8* and *mex-3* function, the number of GSCs is strongly reduced (Ariz et al., 2009). In addition, the function of *puf-8* in inhibiting GSC proliferation was initially discovered in the background of a gain-of-function mutation of the gene *glp-1*, which encodes a *C. elegans* ortholog of human NOTCH. Specifically, the loss of *puf-8* was found to enhance the ability of gain-of-function mutations of *glp-1*^Notch^ to promote germline tumour formation (Racher and Hansen, 2012).

*puf-8* functions in various additional processes. By repressing the expression of the *pal-1* gene, *puf-8* prevents the transcription in the germline of soma-specific genes (Mainpal et al., 2011). Most likely by controlling the expression of components of the germline sex-determination pathway, *puf-8* also contributes to the sperm/oocyte switch in the developing hermaphrodite germline (Bachorik and Kimble, 2005). Furthermore, in developing somatic tissues, *puf-8* is expressed in the six ‘vulval precursor cells’ (VPCs), where it represses the vulval cell fate, possibly by negatively regulating *let-60* (*Ras*) signalling (Walser et al., 2006). *puf-8* mRNA is also detected in the presynaptic compartment of the adult nervous system where PUF-8 protein may contribute to associative learning (Arey et al., 2019). Finally, *puf-8* has been proposed to reduce lifespan by promoting mitochondrial fission and mitophagy in adults (D'Amico et al., 2019). Like the phenotypes of *puf-8* in the context of apoptosis and GSC proliferation, the phenotypes caused by the loss of *puf-8* in these various processes are of low penetrance and often observed only in sensitized genetic backgrounds. Therefore, it will be interesting to see whether *puf-8* also acts to ‘fine-tune’ these processes by balancing opposing activities.

### PUF-8 fine-tunes apoptosis by repressing the expression of *ced-3*, and possibly *ced-4* and *ced-9*

In the hermaphrodite germline, *puf-8* promotes mitosis in GCSs by facilitating *glp-1* signalling. It has been proposed that this is the result of a *puf-8*-dependent increase in the expression of the gene *farl-11*, which encodes a protein that localizes to the endoplasmic reticulum and is required for the association of GLP-1 with membranes (Ariz et al., 2009; Maheshwari et al., 2016). Furthermore, *puf-8* promotes the ability of GSCs to enter meiotic prophase and to differentiate by impeding *let-60* signalling, and it has been proposed that this is the result of a *puf-8*-dependent decrease in *let-60* expression (Racher and Hansen, 2012; Vaid et al., 2013). Indeed, there is evidence that both *farl-11* and *let-60* are direct targets of *puf-8*, and that PUF-8 protein impacts their expression by binding to PBEs in the 3′ UTR of their respective mRNAs (Vaid et al., 2013; Maheshwari et al., 2016).

Our results suggest that, in the context of apoptosis, *puf-8* impacts the expression of components of the central apoptosis pathway. The pro-apoptotic *ced-3* gene contains six PBEs in its 3′ UTR. We present evidence in support of the model that PUF-8 protein binds to these six PBEs and promotes *ced-3* mRNA turnover, thereby repressing *ced-3* expression and preventing the apoptotic death of cells that normally do not die. Similarly, the pro-apoptotic *ced-4* gene contains two PBEs in its 3′ UTR and our results suggest that PUF-8 may also bind to these two PBEs, thereby promoting *ced-4* mRNA turnover. Our results also suggest that PUF-8 may bind to five PBEs in the 3′ UTR of the anti-apoptotic *ced-9* gene and promote *ced-9* mRNA turnover, thereby repressing *ced-9* expression and promoting the apoptotic death of cells that normally die. Alternatively, PUF-8 may impact *ced-4* and *ced-9* mRNA copy numbers indirectly through currently unknown PUF-8 target mRNAs. Furthermore, whether PUF-8 also represses translation of *ced-3* (and *ced-4* and *ced-9*) mRNAs remains to be determined. Interestingly, *ced-3* has previously been suggested to be a target of *puf-8* in the hermaphrodite germline and in GSCs, in particular, where the *puf-8* gene is highly expressed (Subasic et al., 2016). Specifically, it has been shown that the knockdown by RNA-mediated interference (RNAi) of *puf-8* results in an increase in the expression of both a *ced-3::gfp* transgene and a *ced-3* 3′ UTR reporter in GSCs. However, the relevance of this is currently unclear, as knocking down *puf-8* function [*puf-8*(*RNAi*)] failed to cause an observable apoptosis phenotype in the germline (Subasic et al., 2016).

Finally, it has been proposed that the ability of RBPs to impact expression of target mRNAs is connected to the functions of microRNAs that may bind overlapping or adjacent sites in the same target mRNA (van Kouwenhove et al., 2011; Kim et al., 2021). Indeed, *C. elegans* PUF-9 was shown to be required for the ability of the microRNA LET-7 to repress the expression of the gene *hbl-1* (Nolde et al., 2007). We have previously shown that, like *puf-8*, the *mir-35* and *mir-58* families of microRNA genes prevent mothers of cells that are programmed to die from dying inappropriately (Sherrard et al., 2017). However, in that study, we found that miR-35 and miR-58 microRNAs act through microRNA binding sites in the 3′ UTR of the pro-apoptotic gene *egl-1*, not the pro-apoptotic genes *ced-3* or *ced-4*. Based on our analyses, the 3′ UTR of *egl-1* does not contain PBEs, *egl-1* mRNA copy numbers do not significantly change in the RIDnb in animals lacking *puf-8* function, and PUF-8 protein does not physically interact with *egl-1* mRNA *in vivo*. For this reason, we consider it unlikely that the activity of PUF-8 is influenced by miR-35 and/or miR-58 microRNAs in this context.

### The roles of PUF-8, PUM1 and PUM2 in robustness and cell number homeostasis

Controlling the number of cells is crucial for the development of multi-cellular animals and for the maintenance of cell number homeostasis throughout adult life. How cell numbers are controlled is not well understood. Based on previous studies and the results described here, we propose that *C. elegans* PUF-8 plays a crucial role in developmental robustness and cell number homeostasis in both the germline and somatic tissues. In the germline, *puf-8* activity is required to balance proliferation and differentiation in germline stem cells (GSCs), thereby maintaining germline homeostasis (Wang and Voronina, 2020). Similarly, in somatic tissues, *puf-8* activity is required to balance life and death, thereby maintaining the fidelity of the highly reproducible pattern of cell survival and cell death during development. In both tissues, the loss of *puf-8* results in susceptibility to perturbations such as stochastic differences in gene expression or variations in genetic background.

Interestingly, as a result of p53-dependent apoptosis, the loss of PUM1 in mice causes a reduction in the number of spermatocytes and testicular hypotrophy (Chen et al., 2012). Furthermore, the human *Pum1* and *Pum2* genes have been shown to be mis-expressed in various types of cancers, and there is increasing evidence that this misexpression impacts tumorigenesis. For example, it has recently been demonstrated that in human colorectal cancer (CRC), *Pum1* and *Pum2* are expressed at elevated levels and that the knockdown by siRNA of *Pum1* and *Pum2* in a mouse model of CRC inhibits tumour progression (Goldstrohm et al., 2018; Smialek et al., 2021; Gong et al., 2022). This suggests that the roles of PUF-8, and PUM1- and PUM2-like RBPs in the control of apoptosis and cell number homeostasis may be conserved.

## MATERIALS AND METHODS

### General *C. elegans* strain maintenance and alleles

All *C. elegans* strains used in this study are listed in Table S1 and were cultured and maintained as described by Brenner (1974). Animals were grown at 20°C on nematode growth medium (NGM) plates with *E. coli* OP50 bacterial lawns. Experiments were conducted at 25°C, unless stated otherwise. The Bristol N2 strain was used as wild type, and the following transgenes and alleles were used in this study: LGII: *puf-8(q725)* (Bachorik and Kimble, 2005), *puf-8(ok302)* (Subramaniam and Seydoux, 2003), *fbf-1(ok91)fbf-2(q704)/mIn1[dpy-10(e128) mls14]* (Crittenden et al., 2002) and *puf-8(syb2309) (3xFLAG::puf-8)* (this study; made by SunyBiotech); LGIII: *ced-9(n1653ts)* (Hengartner et al., 1992) and *unc-119(ed3)* (Maduro and Pilgrim, 1995); LGIV: *bcSi87* (P*_puf-8_ puf-8*) (this study), *ced-3(n717)* (Ellis and Horvitz, 1986), *ced-3(n2427)* (Shaham et al., 1999) and *ced-3(bc448)* (this study); LGV: *egl-1(n3330)* (Sherrard et al., 2017); LGX: *puf-9(ok1136)* (Nolde et al., 2007). In addition, the following multicopy transgenes and extra-chromosomal arrays were used: *wgIs675 (atfs-1::TY1::EGFP::3xFLAG*) (Sarov et al., 2006; Zhong et al., 2010) and *xdEx1091* (P*_unc-3_unc-3::gfp*+P*_sur-5_rfp*) (Wang et al., 2015). Animals of the genotype *puf-8(q725); ced-9(n1653ts)* used in this study were the homozygous F1 progeny of *puf-8(q725)/mIn1; ced-9(n1653ts)* hermaphrodites.

### Plasmid construction

The primers used in this study are listed in Table S2 and plasmids generated are listed in Table S3. The plasmid pBC1815 was constructed using two-step overlap extension PCR and restriction enzyme cloning. First, a 3868 bp fragment covering the *puf-8* locus was amplified from N2 lysates using primers *puf-8* locus F and *puf-8* locus R. The resulting PCR fragment was cloned into MosSCI vector pCFJ350 between the AflII and SpeI sites using T4 DNA ligase. The plasmid pBC1816 was constructed using Gibson cloning. The *puf-8* promoter (1624 bp) was amplified from wild-type genomic DNA using primers *puf-8* promoter F and *puf-8* promoter R, the *C30G12.6* transcription unit (genomic fragment) was amplified using primers *C30G12.6* F and *C30G12.6* R, and the MosSCI vector pCFJ350 was amplified with primers pCFJ350 F and pCFJ350 R. The three PCR fragments were assembled using Gibson cloning.

### Generation of transgenic strains

The single-copy integrations *bcSi86* and *bcSi87* were generated using universal MosSCI (Frøkjaer-Jensen et al., 2008) with the plasmids pBC1816 and pBC1815, respectively. The universal MosSCI strain EG8081 [*oxTi177; unc-119(ed3)*] was used for targeted insertion on LGIV. To generate MosSCI strains, plasmids pBC1816 and pBC1815 were separately injected into the MosSCI strains at a concentration of 10 ng/µl along with the co-injection plasmids pCFJ601 at 50 ng/µl, pGH8 at 10 ng/µl, pCFJ90 at 2.5 ng/µl and pCFJ104 at 5 ng/µl.

### CRISPR/Cas-mediated genome editing

*ced-3(bc448)* was generated using CRISPR/Cas12a(Cpf1) genome editing technology (Zetsche et al., 2015). Small guide RNAs targeting the *ced-*3 gene were designed by CRISPOR (Concordet and Haeussler, 2018). Two sgRNAs (from IDT) were used to target the upstream region and downstream region of *ced-3* 3′ UTR (guide sequences: 5′-GCCGGAAGCACGAAACTCTGCCG-3′ and 5′-TTCGATTCCTCCTCTCCGCGCAC-3′, respectively). A 909 nt single strand DNA (ssDNA) donor that carries the PBE-mutated *ced-3* 3′ UTR fragment and 38 nt homology repair sequence was used. To prepare this ssDNA donor, the PBE-mutated *ced-3* 3′ UTR DNA fragment with homology repair sequence was first prepared. Briefly, overlapping PCRs were first performed to change the first three nucleotides from TGT to CCC in each of the six PBEs identified in the *ced-3* 3′ UTR DNA fragment. This PBE-mutated *ced-3* 3′ UTR fragment was inserted into backbone pCFJ350, generating the plasmid pBC1893. After this, the PBE-mutated *ced-3* 3′ UTR fragment was amplified from plasmid pBC1893 using oligo oYJ145 and oYJ146 (Table S2), through which the homology repair sequence was added. The resulting PBE-mutated *ced-3* 3′ UTR fragment with homology repair sequence was used as the template for asymmetric PCR to prepare the ssDNA donor by primer oYJ146. The microinjection of Cas12a(Cpf1)-sgRNA ribonucleoproteins together with the ssDNA donor and screening for genome editing were performed as previously described (Ghanta et al., 2021). The lines generated were confirmed by sequencing.

### 4D microscopy and lineage analysis of embryonic cell death

L4 larvae were grown to the adult stage overnight at 25°C. Two- or four-cell embryos were collected from young adults, mounted on 2% agarose pads, covered with a coverslip, and the coverslip sealed with Vaseline. 4D recordings were made throughout embryonic development as described previously using a Zeiss Axio Imager M2 and ‘Time to Live’ software (Caenotec) (Schnabel et al., 1997, 2006). Each recording captures 25 DIC *z*-slices (*z*-step 1 µm; from the top to the bottom of the embryo) every 35 s at 25°C. The entire recording time was 7 h. Lineaging analysis was performed using ‘Simi BioCell’ software (Simi Reality Motion Systems). The number of corpses per embryo was scored until ventral enclosure. The cell deaths were identified by cell lineaging. The ‘time to form corpse’ was determined by measuring the time (in min) from post-cytokinesis to the formation of a refractile cell corpse.

### Embryonic lethality assay

Individual hermaphrodites at the L4 stage of development were cultured on 35 mm NGM plates containing OP50. Each hermaphrodite was allowed to lay embryos for 12 h at 20°C. Adult hermaphrodites were then transferred to fresh plates and again allowed to lay embryos for 12 h. This was repeated until the hermaphrodites no longer produced embryos. The number of embryos laid on each plate was counted once the hermaphrodites had been removed. The number of dead embryos on each plate was counted after 24 h. For each genotype, the total number of embryos is the sum of the numbers of embryos laid on each plate. The total number of dead embryos is the sum of the numbers of dead embryos on each plate. Ultimately, embryonic lethality was calculated as the percentage of dead embryos among all embryos laid.

For the percent embryonic lethality data shown in Fig. 1E, the numbers of embryos analyzed (*n*) were 2940 for +/+, 2411 for *ced-3(n717)*, 785 for *ced-9(n1653ts)*, 3307 for *puf-8(q725)*, 2947 for *ced-9(n1653ts); ced-3(n717)*; 415 for *puf-8(q725); ced-9(n1653ts)*, 3272 for *puf-8(q725); ced-3(n717)* and 1457 for *puf-8(q725); ced-9(n1653ts); ced-3(n717).* For the percent embryonic lethality data shown in Fig. 5D, the numbers of embryos analyzed (*n*) were 323 for *+/+*, 343 for *ced-3(bc448)*, 426 for *ced-9(n1653ts)*, 412 for *ced-9(n1653ts);ced-3(bc448)*, 154 for *puf-8(q725);ced-9(n1653ts)* and 206 for *puf-8(q725);ced-9(n1653ts);ced-3(bc448)*.

### Analysis of precocious RIDnb death and inappropriate RIDsc survival

The RIDnb divides and gives rise to two daughter cells at the bean stage of embryonic development: the RID, which survives and forms the RID neuron, and the RIDsc, which dies 20-30 min after the completion of RIDnb division (around the comma stage). To score precocious RIDnb death and inappropriate RIDsc survival in *puf-8* loss-of-function mutants, embryos were isolated from gravid adults, mounted on 2% agarose pads and incubated at 20°C for 4 h (wild type) or 5 h [*puf-8(q725)* mutants] to allow the embryos to develop to the bean stage. RIDnb and its daughters were identified using a P*_unc-3_unc-3::gfp+P_sur-5_rfp* transgene (*xdEx1091*) (Wang et al., 2015), which is expressed in all three cells, and a Zeiss Axio Imager M2 with both DIC and GFP channels. We observed three possible outcomes:

(1) Two GFP positive cells were generated at the anterior side of the embryo and one of these cells turned into a refractile cell corpse at the comma stage. This outcome means that the RIDnb survived and divided, and that the RIDsc died.

(2) One large GFP-positive cell corpse was observed at the bean stage. This outcome means that the RIDnb died precociously.

(3) Two GFP-positive cells were generated at the anterior side of the embryos but neither one formed a retractile cell corpse until the completion of the recordings (two-fold stage). This outcome means that the death of the RIDsc was either delayed or blocked (inappropriate survival).

### Single-molecule RNA fluorescent *in situ* hybridization

Single-molecule RNA fluorescent *in situ* hybridization (smRNA FISH) was performed in *C. elegans* embryos as described (Raj et al., 2008; Sherrard et al., 2017) with slight modifications. Stellaris FISH probes (Biosearch Technologies) labelled with TAMRA or Quasar-670 were designed against the mature mRNAs of *egl-1*, *ced-9*, *ced-4*, *ced-3* or *unc-3::gfp*. Eight healthy L4 worms were transferred to a medium NGM plate and grown at 20°C for 4 or 5 days until the plate was full of adults. On the first day, adults were then harvested with M9 buffer and embryos collected in a 2 ml Eppendorf tube by dissolving adults in a solution containing bleach (0.6% NaHOCl and 0.7N NaOH in nuclease-free water). The embryos were cultured in M9 buffer at 25°C for 2.5 h so that they could reach the desired stage of development. Embryos were then pelleted and resuspended in fixation solution (3.7% formaldehyde and 1×PBS in nuclease-free water) and incubated on a rotator for 15 min at room temperature. The tube was immediately vortexed and submerged in liquid nitrogen for 1 min to freeze crack eggshells. The tube was thawed in water at room temperature, vortexed and placed on ice for 20 min. Embryos were washed twice with 1 ml of 1×PBS, resuspended in 70% ethanol to permeabilize membranes and kept rotating at 4°C overnight. The following day, embryos were pelleted, resuspended in 100 µl hybridization buffer (10% dextran sulfate, 1 mg/ml ribonucleic acid, 2 mM ribonucleoside vanadyl complex, 200 µg/ml BSA, 10% deionized formamide and 2×SSC in nuclease-free water) supplemented with desired FISH Probes and incubated in the dark at 30°C overnight. On the third day, embryos were resuspended in 1 ml wash buffer twice at 30°C for 30 min. Embryos were then pelleted and resuspended in wash buffer supplemented with 5 ng/ml DAPI for nuclear counterstaining, incubated at 30°C for 30 mins, and resuspended in 10-30 µl VECTASHIELD antifade mounting medium. The amount of antifade mounting medium depended on the final number of embryos.

For imaging, embryos were briefly vortexed to resuspend and 1.5 µl Vectashield with embryos was applied to a round glass cover slip. A square cover slip was put on top of the drop, effectively sandwiching the embryos between the two glass surfaces. Next, a square silicon isolator was adhered to a standard glass microscope slide. The prepared square cover slip was adhered on top of the silicon isolator, such that the round coverslip was hanging upside-down inside the airtight chamber made by the glass slide, the silicon isolator and the square cover slip. Imaging was performed using a Leica TCS SP8 confocal microscope with a 63× oil immersion lens and a *z*-step size of 0.5 µm to capture diffraction-limited spots over several *z*-slices. Leica LAS AF software was used to capture images with constant settings of 512×512 pixels, 600 Hz and a line averaging of 3. The sequences of the smRNA FISH probes used throughout this study are listed in Tables S4-S8.

### Quantification of mRNA copy number in the RID lineage

To quantify mRNA copy numbers in cells of interest, image analysis was performed using Fiji software as described previously (Sherrard et al., 2017). The RIDnb and its two daughter cells can be detected using the reporter *xdEx1091* (P*_unc-3_unc-3::gfp*+P*_sur-5_rfp*) (Wang et al., 2015) in the GFP channel. Briefly, a three-dimensional region of interest (ROI) was defined for the cells of interest or an entire embryo. All individual *z*-slices for this ROI were summed through the *z*-projection. The smRNA FISH signal intensity was measured with ‘Analyse-Measure-Integrated Density’ in Fiji. The signal intensity of the background was measured in the same way in cells that had no mRNA signal. The smRNA FISH signal intensity minus the average of the signal intensity of three backgrounds equals the final mRNA signal intensity. The final intensity of single mRNAs was the average intensity of three single mRNAs minus the intensity of three backgrounds. Finally, mRNA copy number for the cell of interest was calculated by dividing the final smRNA FISH signal intensity of the ROI by the final intensity of a single mRNA. For the purpose of presentation in figures, maximum intensity *z*-projection images were smoothened (Gaussian blur: radius, 1.2) and the DAPI signal of neighbouring nuclei was removed.

### Immunoprecipitations and western blots

Immunoprecipitations (IPs) were performed as previously described with the following modifications (Kershner and Kimble, 2010; Shin et al., 2017). Strains homozygous for *3xFLAG::puf-8* [*puf-8(syb2309)*] or *atfs-1::EGFP::3xFLAG* (*wgIs675*) were cultivated on 100 mm NGM plates at 20°C until they had generated a lot of young adults. Animals were harvested and washed three times with M9 buffer [3 g/l KH_2_PO_4_, 6 g/l NaHPO_4_, 5 g/l NaCl and 1 mM MgSO_4_]. Worm pellets were aliquoted (100 mg/tube), snap frozen in liquid nitrogen, resuspended in 500 μl lysis buffer [20 mM Tris HCl (pH 7.5), 150 mM NaCl, 1 mM EDTA, 0.1% NP-40, 1× cOmplete Protease Inhibitor cocktail (Roche, 05892791001) and 200 U/ml SUPERase In RNase Inhibitor (Invitrogen, AM2694)] and homogenized through sonication (amplitude 40%, pulse on 5 s, pulse off 30 s, 10 cycles). To remove insoluble material, worm lysates were centrifuged at 15,000 ***g*** for 10 min at 4°C. To prepare antibody-coated beads, 10 μg anti-FLAG (M2 clone, Sigma F1804) was incubated with 3 mg protein G Dynabeads (Invitrogen 10007D) for 30 min at room temperature. Next, 400 μl aliquots of lysate were incubated with the antibody-bead mixture for 2 h at 4°C on a rotating platform. The beads were pelleted and washed four times with the washing buffer included in the kit (Invitrogen, 10007D). To elute bound proteins, 10% of IP beads were heated at 90°C for 10 min in 2×SDS/PAGE sample buffer. RNA was eluted from remaining beads using 500 μl TRIzol reagent (Invitrogen, 15596026).

The proteins precipitated were analyzed by western blotting. Protein samples equivalent to 1% of the input or the immunoprecipitation (IP) IP were loaded into each well of a 10% sodium dodecyl sulfate (SDS)–polyacrylamide gel. After SDS-PAGE, the proteins were transferred to a polyvinylidene fluoride (PVDF) membrane and blocked using a blocking buffer (Bio-Rad, 12010020). Blots were incubated overnight at 4˚C with either Mouse anti-FLAG antibodies (M2 clone, Sigma F1) or mouse anti-actin antibodies (C4 clone, Sigma, MAB1501) at a dilution of 1:2000. After washing, blots were incubated at room temperature for 1 h with a Light-Chain Specific Goat Anti-Mouse IgG (conjugated with HRP, Cell Signal Technology, 91196) at a dilution of 1:5000. Immunoblots were developed using an ECL Select Western Blotting Detection Reagent (Sigma, GERPN2235) and imaged using an iBright FL1500 Imaging System (Invitrogen).

### Reverse transcription and quantitative PCR

The RNA from the aqueous phase was pelleted using isopropanol and 5-10 μg GlycoBlue Coprecipitant (Invitrogen, AM9515). The RNA pellet was washed using 70% ethanol and dissolved in 30 μl RNase-free water. cDNA was prepared from 10 μl input and IP RNA samples using the M-MLV Reverse Transcriptase (Invitrogen, 28025013). cDNA was analyzed using SYBR Green PCR Master Mix (Invitrogen, 4344463) on a QuantStudio 3 Real-Time PCR System (Thermo Fisher Scientific). *tbg-1* was used as the endogenous control for normalization, and data were analyzed using the ΔΔCT method (Schmittgen and Livak, 2008). Occasionally, CT readings in the IP samples were undetectable and were set to the maximum cycle number. Enrichment of mRNAs present in 3xFLAG::PUF-8 IP was compared with ATFS-1::EGFP::3xFLAG IP. The primers used for the qPCR are listed in Table S2.

### Statistical analyses

The number of embryos or cells analyzed (*n*) is stated in the figure legends. Graph preparation and statistical analyses were performed using GraphPad Prism (GraphPad Software). Unpaired *t*-tests and one-way ANOVA were used for the parametric tests of two and multiple groups, respectively. Mann–Whitney test was used as an alternative non-parametric test. Fisher's exact test was used for contingency tests.

## Supplementary Material

10.1242/develop.201167_sup1 Supplementary information
