# Supplementary material for: PUF-8, a C. elegans ortholog of the RNA-binding proteins PUM1 and PUM2, is required for robustness of the cell death fate
Source: Development. 2023 Oct 6;150(19):dev201167. doi: 10.1242/dev.201167 (PMC10565243; doi:10.1242/dev.201167)
Supplement: Supplementary information [file develop-150-201167-s1.pdf]

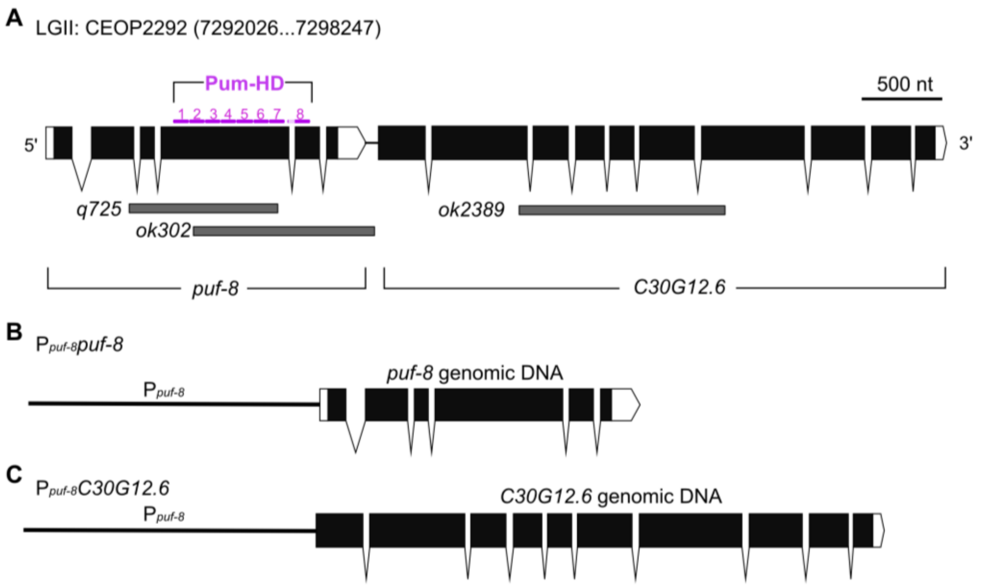

Fig. S1. Schematics of operon CEOP2292 and *puf-8*$^{\mathrm{PUM1,\ 2}}$ gene.

(A) Schematic of operon CEOP2292 on chromosome II (LGII), which contains the genes *puf-8* and *C30G12.6*. *puf-8* is 2138 nt in length and resides on the negative strand. Black boxes represent exons, while connecting lines represent introns; white boxes represent UTRs. The Pum-HD including its eight Puf repeats (in pink) are shown. Regions deleted by the strong loss-of-function alleles *q725, ok302* and *ok2389* are indicated in grey. (B) Schematic of rescuing transgene $P_{puf\text{-}8}$*puf-8* (*bcSi87*). The *puf-8* transcription unit is driven by the *puf-8* promoter (1624 bp). (C) Schematic of transgene $P_{puf\text{-}8}$*C30G12.6* (*bcSi86*). The *C30G12.6* transcription unit is driven by the *puf-8* promoter (1624 bp).

A

Genotypes	Large corpses (#)	n
+/+	0	6
puf-8(q725)	1.4±0.9	14
puf-8(ok302)	1.3±0.9	4
C30G12.6(ok2389)	0	10
puf-8(+); puf-8(q725)	0	5
puf-8(q725); C30G12.6(+)	1.4±0.2	10

B

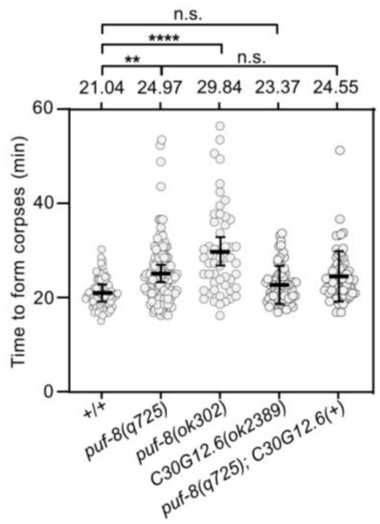

Fig. S2. The loss of *C30G12.6* does not result in inappropriate death or survival.

(A) Average numbers of large corpses per embryo during embryogenesis before ventral enclosure in different genotypes. +/+ indicates wild type. The number of embryos examined is given (n). (B) The time to form a corpse was measured in minutes for cell deaths of the 1st wave in the genotypes indicated. Grey dots represent the values for individual cell deaths. Average time to form corpse are given above each data set. Error bars indicate the average ± SD. (∗) P ≤ 0.05; (∗∗) P ≤ 0.01; (∗∗∗∗) P ≤ 0.0001 via two-tailed Mann-Whitney test.

A

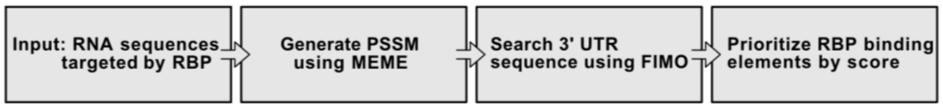

B

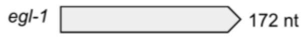

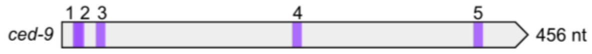

Element	Sequence	Conservation
ced-9-1	UGUGUAAA	-
ced-9-2	UGUAAAUA	*bre, bri, jap, rem*
ced-9-3	UGUACAAC	*bre, rem*
ced-9-4	UGUACAUU	-
ced-9-5	UGUAUUUA	*bre, bri, rem*

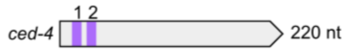

Element	Sequence	Conservation
ced-4-1	UGUACAAU	*bre, bri, rem*
ced-4-2	UGUAUAUU	-

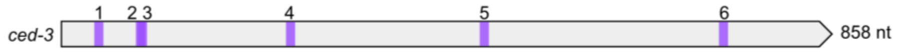

Element	Sequence	Conservation
ced-3-1	UGUCUGUA	-
ced-3-2	UGUGUAUA	*bre, bri, jap, rem*
ced-3-3	UGUAUAUU	*bre, bri, jap*
ced-3-4	UGUAUGUA	*jap, rem*
ced-3-5	UGUAUAAA	*bre, bri, jap, rem*
ced-3-6	UGUACGUU	*bre, bri, rem*

Fig. S3. Identification of PUF-8$^{PUM1, 2}$ binding elements in 3′ UTR sequences.

(A) Pipeline for identification of PUF-8 binding elements (PBEs) in 3′ UTRs of cell death genes. RNA sequences bound by PUF-8, as determined by Opperman et al. 2005, were used to generate PSSMs using MEME. These matrices were subsequently used by FIMO to search 3′ UTR sequences for potential elements, which were ranked according to the log-odds score output by FIMO. (B) Schematics and sequences of predicted PUF-8 binding elements (PBEs) in 3′ UTRs of *egl-1*, *ced-9*, *ced-4* and *ced-3*. Conservation in related *Caenorhabditis* species is indicated (*bre – C. brenneri; bri – C. briggsae; jap – C. japonica; rem – C. remanei*) The purple boxes represent the sites of potential PBEs. There is no PBE in *egl-1* 3′ UTR. There are five, two and six PBEs in 3′ UTRs of *ced-9*, *ced-4* and *ced-3*, respectively.

Development • Supplementary information

Table S1. List of strains.

Strain name	Genotype	Source
N2	Wild type	(Brenner 1974)
JH1521	puf-8(ok302) II	(Subramaniam and Seydoux 2003)
JK3107	fbf-1(ok91) fbf-2(q704)/mIn1 [dpy-10(e128) mIs14] II	(Crittenden et al. 2002)
JK3231	puf-8(q725) II	(Bachorik and Kimble 2005)
MD131	egl-1(n3330) V	This study
MD2175	ced-9(n1653ts) III	This study
MD3887	xdEx1091 (P_{unc-3}unc-3::gfp + P_{sur-5}rfp)	This study
MD4025	puf-8(q725) II; puf-9(ok1136) X	This study
MD4048	puf-8(q725) II; egl-1(n3330) V	This study
MD4055	puf-8(q725) II; ced-3(n717) IV	This study
MD4351	C30G12.6(ok2389) II	This study
MD4373	puf-8(q725) II; ced-3(n2427) IV	This study
MD4486	puf-8(q725) II; bcSi87 [P_{puf-8} puf-8 + unc-119(+)] IV	This study
MD4495	puf-8(q725) II; bcSi86 [P_{puf-8} C30G12.6 + unc-119(+)] IV	This study
MD4639	puf-8(q725) II; xdEx1091 [P_{unc-3}unc-3::gfp + P_{sur-5}rfp]	This study
MD4640	puf-8(ok302) II; xdEx1091 [P_{unc-3}unc-3::gfp + P_{sur-5}rfp]	This study
MD4644	puf-8(q725)/mIn1 II; ced-9(n1653ts) III	This study
MD4668	puf-8(q725) II; ced-9(n1653ts) III; ced-3(n717) IV	This study
MD4682	puf-8(q725) II; ltIs44 [P_{pie-1}mCherry::PH(PLC1^{delta1}) + unc-119(+)] V; xdEx1091 [P_{unc-3}unc-3::gfp + P_{sur-5}rfp]	This study
MD4683	ltIs44 [P_{pie-1}mCherry::PH(PLC1^{delta1}) + unc-119(+)] V; xdEx1091 [P_{unc-3}unc-3::gfp + P_{sur-5}rfp]	This study
MD4834	ced-3(bc448) IV	This study
MD4845	ced-9(n1653ts) III; ced-3(bc448) IV	This study
MD4853	puf-8(q725)/mIn1 II; ced-9(n1653ts) III; ced-3(bc448) IV	This study
MT1522	ced-3(n717) IV	(Ellis and Horvitz 1986)
MT7004	ced-3(n2427) IV	(Shaham et al. 1999)
MT7536	ced-9(n1653ts) III; ced-3(n717) IV	(Hengartner et al. 1992)
OP675	unc-119(tm4063) III; wgIs675 [atfs-1::TY1::EGFP::3xFLAG]	(Sarov et al. 2006; Zhong et al. 2010)
PHX2309	puf-8(syb2309) II	This study
VC894	puf-9(ok1136) X	(Nolde et al. 2007)

Development • Supplementary information

Table S2. List of primers. The sequences and primer names as well as their applications are listed in the table.

Primer name	Sequence (5′-3′)	Notes
puf-8 locus F	GACTCACTTAAGCAATCGAGAATAGAATGACAAAC	used for plasmid pBC1815 construction
puf-8 locus R	CGACTCACTAGTCTCAAACATTTAGATTTGTGC	used for plasmid pBC1815 construction
puf-8 promoter F	AGGGTACCAGAGCTCACCTAATGACAAACTCTTTGAATGC	used for plasmid pBC1816 construction
puf-8 promoter R	CAGACTCCATCTAAAATAATTACTTTAGGTCAAGG	used for plasmid pBC1816 construction
C30G12.6 F	AATTATTTTAGATGGAGTCTGGTGATCATC	used for plasmid pBC1816 construction
C30G12.6 R	CGTCACCGGTTCTAGATACCTTTGAATAAAAAGGGATTTTATGAAATTAAAC	used for plasmid pBC1816 construction
pCFJ350 F	TAGGTGAGCTCTGGTACCCT	used for plasmid pBC1816 construction
pCFJ350 R	GGTATCTAGAACCGGTGACG	used for plasmid pBC1816 construction
oYJ145	CTCAAAAAGTTCTACTTTTGGCCGGAAGCACGAAACTCTGCCGTCTAAAATTCACTCGTGATTCATTGC	Used for ced-3 ssDNA donor preparation
oYJ146	TTATAATAACAAGCTATATAAATCTCAAC	Used for ced-3 ssDNA donor preparation
tbg-1 F	TGATGACTGTCCACGTTGGA	used for qPCR
tbg-1 R	CGTCATCAGCCTGGTAGAACA	used for qPCR
egl-1 F	GATTCTTCTCAATTTGCCGACG	used for qPCR
egl-1 R	TCATCTGAGCATCGAAGTCATC	used for qPCR
ced-9 F	CTGTATCAGGATGTGGTTCGG	used for qPCR
ced-9 R	AGCGATGTGTAAACGAAGAGG	used for qPCR
ced-4 F	ACGCTTATGATGTTTTTCAAGTCT	used for qPCR
ced-4 R	CCTCATCTGACAAAACTTCAACAC	used for qPCR
ced-3 F	CCAATTTGTTCAGATGCATGGG	used for qPCR
ced-3 R	TCTCCGTGTGATTCGTGTTTG	used for qPCR

Development • Supplementary information

Table S3. List of plasmids.

Plasmid name	Information	Source
pBC1815	$P_{puf\text{-}8}$ puf-8 + unc-119(+)	This study
pBC1816	$P_{puf\text{-}8}$ C30G12.6 + unc-119(+)	This study
pBC1893	$P_{mai\text{-}2}$gfp::h2b::ced-3 3′ UTR $^{6\ PBE}_{mut}$	This study

Table S4. Sequences of 48 probes used for *unc-3::gfp* smRNA FISH (fluorescein labelled).

Probes 1–16 (5′ to 3′)	Probes 17–32 (5′ to 3′)	Probes 33–48 (5′ to 3′)
CATTTTTTCTGAGCTCGGTA	GTGTCCAAGAATGTTTCCAT	CGAGATGTCTGATGACAGCG
CTTACGCTTCTTCTTTGGAG	GTGAGTTATAGTTGTATTCC	GGACTTAGAAGTCAGAGGCA
TACTCATTTTTTCTACCGGT	GTCTGCCATGATGTATACAT	AGAGCATGTAGGGATGTTGA
CCAGTGAAAAGTTCTTCTCC	CTTTGATTCCATTCTTTTGT	AAAATAGGGGGTGGGAGCAC
CCCATTAACATCACCATCTA	CCATCTTCAATGTTGTGTCT	TTTCATTGTTAGAGGTGACT
CCTCTCCACTGACAGAAAAT	ATGGTCTGCTAGTTGAACGC	GGGAGGGAGCACAATTTTTT
GTAAGTTTTCCGTATGTTGC	CGCCAATTGGAGTATTTTGT	GTGTACACAGAACATTGTGT
GTAGTTTTCCAGTAGTGCAA	GTCTGGTAAAAGGACAGGGC	GTATTTTGTGTGCGGTTTTT
ACAAGTGTTGGCCATGGAAC	AAGGGCAGATTGTGTGGACA	GCGGTCATAAACTGAAACGT
GGTATCTCGAGAAGCATTGA	TCTTTTCGTTGGGATCTTTC	CCCAGACGTGCGAAGAAATA
TCATGCCGTTTCATATGATC	TCAAGAAGGACCATGTGGTC	CGATGAGCATGATTTGACGT
GGGCATGGCACTCTTGAAAA	AATCCCAGCAGCTGTTACAA	GGGGAAACCCCAAAAAGCAA
TTCTTTCCTGTACATAACCT	TATAGTTCATCCATGCCATG	AAGAAAAACGCCGTCCTCGA
GTTCCCGTCATCTTTGAAAA	GCTCAGTTGGAATTCTACGA	TGCATCGTGCTCATCAATAC
TGACTTCAGCACGTGTCTTG	CAAGTTGGTAATGGTAGCGA	CTCAAACCCAAACCTTCTTC
TAACAAGGGTATCACCTTCA	CCCTATTATTTTTGACACCA	ATCAACTTCTACTCACCTTC

Table S5. Sequences of 23 probes used for *egl-1* smRNA FISH (TAMRA labelled).

Probes 1–8 (5′ to 3′)	Probes 9–16 (5′ to 3′)	Probes 17–23 (5′ to 3′)
CGGTGTGAATGTTTTGGGTG	AAGAATCTTCACACGAGGAG	GTTGGAGATTTTGATCACTT
AAGAGAAGTTAGAATACGAC	AAAAAATCCCGAGTCGTCGG	CATGGTACAAATTGGAGAA
AGCATCAGCATATCAACTGA	CGATGCTGCTGATCTCAGAG	CACCGGGTATTATGAGAA
ATCCGAAGAGGTTGAGGCAA	ATTGCTGCTAGCTTGGAGCC	TAATCACAAATGAAGAAAA
AAACGTTGGACATTGGTAGA	GAGCATCGAAGTCATCGC	TGGAGACGGAGAGATCGAAA
AAAACGGAAGATTGAACGTC	TGGGCCGAGTAGGACATCAT	TGGTACAAATATTGAGGGGA
ACATGTTCTTTTCGTTGT	GAAGAGGCTTCTGTCGGAAG	AATATGAGCAATAAAGGAC
GTCCTGAGACGAGGAGTA	GCGAAAAAGTCCAGAAGACG	

Table S6. Sequences of 44 probes used for *ced-9* smRNA FISH (Quasar 670 labelled).

Probes 1–15 (5′ to 3′)	Probes 16–30 (5′ to 3′)	Probes 32–44 (5′ to 3′)
GATTCGTCAGCGAGTTGTC	TTCTCGAATATCGTTCCCA	ACTTTTTCAGCTTCTGCTC
CATCGTTCGTCGCCGATAC	CTCAAAATTTTCCGCGTGC	AATCATCGACCACCGTCTG
TTTTATCCCCAGAAACTCC	AGCAGCTGCTCACAGAAGG	CAATGGCTCCAGCTGTTAC
CAAAATCGGTGGGCTCTGT	ATGAGATTCTGGGCACTGC	CACACGACGACTCCAACGA
CCTGAGCATCACTATTGAT	CCACATCCTGATACAGTGA	CAAGCTGAACATCATCCGC
CCTACTCGGTGATGGCAAG	CTGTCTGTGCATTTCCAAC	CCACTTCTTCCAGTTCAAA
CATTCTTCGCGTCGAAGCC	CGTCCATAAGACATTGGAC	GTAATTTGTGGCCTAGCTT
TCAATTGACTCTCCGATGG	CCGAACGAGATTAGACCTA	ACGCGGAAGTAGGGTTTGG
CCCAATCATTGATTTTTCC	TTTTTGCAGCTACGAAACC	GGGTTTCAGGGTTTTTGAC
CGATATCAAGCCTTGGCTC	CAGTTCCACGGATTCCATC	TAGGCCACGGCGAGAAAAG
ATAGTCGACCACAAATCCC	GAAGAGGTTTCGCACTTGT	GTGGAAGAGAAGCGGGAGG
CGTTTTGCCGGATTCGGTG	CCGCGTTTTGATGAACAGC	GATACAGGGGTACTTTGGA
TGCTCCAAACCATTCCATT	ATTGTGTTCCTTCCAGTTG	GAGTTGGAAGAGGCCACAC
TTGCACTCCACACGGCAAT	GAGTGTCATGAAGTCGTCC	CGCGTACAGGAATTTGGGG
AACTCGCATCATTTCGTGC	TTCGTAGTCCTCTTTCATT	

Development • Supplementary information

Table S7. Sequences of 48 probes used for *ced-4* smRNA FISH (Quasar 670 labelled).

Probes 1–16 (5′ to 3′)	Probes 17–32 (5′ to 3′)	Probes 33–48 (5′ to 3′)
CATTCGATTTCGCAGAGCAT	CCACTATCTTTGAGCCAAAC	CATCTTTGAAGAGCCATTGC
TCACGTGGTTCAAAGTCGTG	TCTTCGCTTTTTAGCATCAG	CTTCGATCCTCATCTGACAA
AAAATTGGCGATCCTCTCGA	TCCACCGATGGGAAATTGAG	GCATCACAACTGCGAAAGCA
GCTTGACGTCGATAGATTCG	GCAGATCATCCTTTTGAGTA	GCTTGACGGGTATATCAACT
AAGTCGATGAGTGGTCCAAG	TTGGACGATCAATGAGTGCG	CGCAACTTCATCATCCAATT
GCAAGGTGACTTTGATTGTT	CCCAACGAATTGTTTCTTCT	TTTGCTGAGTCTTTTCAACC
ATCGATGTAGTCTTCGAGGA	AGACATCGAAGACGTAGCTC	AAAACGGGCATTCGTTTTCC
GACGAAGTAGATCTGGCTCA	TATTTCCACGTCACGAGTAG	TGCATCAACGACGTGTTTCA
GTCGGGAAAATTGTGGAGCA	GCATGTTTGAGAAGCAGCAT	ATTGAGATTCCATTGGCGAT
CAATAGTTTCCTATCGAGCA	CGATTTCCAATGATGTCACT	TTCAAGAAGACGCTGCTCGA
TTTGTTTTGGAACATTCCCA	GCATTCCATAAGCTTCTAGA	TGTGATGGTATATGTCGCTC
TCCACGTGATACTCTCGAAT	TCTTCTTTTTCTCCAACAGG	CTGGCTGATGAACGACGGAA
TCGTCGAGCTTTTTGATCAC	CACTGCTTAGTTCGATTGTT	TCACAGTTTCTTCTGTAGTT
TCGGCCGTGTAGAAACAGAA	CATCATAAGCGTTGCTGGAT	TTTGGGAAGTCTTCAGGACG
ATTACTGATTTTCCGGATCC	ACTAATCCTCGACTTTCCAA	TTTCTGGTGCAATTGCATGA
GGTCAGATTTCGAAAGAGCT	CTTGTACGAGTAAGGGGTGA	TCGAGGGGAATATACAGGCA

Table S8. Sequences of 48 probes used for *ced-3* smRNA FISH (TAMRA labelled).

Probes 1–16 (5’ to 3’)	Probes 17–32 (5’ to 3’)	Probes 33–48 (5’ to 3’)
CTTCTATCTTGACGCATCAT	TATTGAGTTGGTCCAGAAGC	CTCGACAAGCCTGCACAAAA
CATAATGTTCCTCTCTAGCA	TCATATCCTCTTCATGGAAT	AATCCATTGTCACGACGTTC
ACACTTGTTTTGCGATGAGA	ATGGTTGGTGCATCGACAAA	AGGAACTCCGTCGACAGAAT
CGAACCGTTCCACATGAATT	TTTCTCGTCGAAAACACGGC	CCCATCCACGACGAAGAAAT
CATAAAACGCGTCGAACGCC	AGGACTCGAGAAGTTTCTGT	AAATTGAACAATGGCCCGTC
GGCGAGAGGTTCAAGAACTT	TTATGATGAGGCACATTCCA	TTTCTCCACACTTGCTGAAC
CGACAGCATTCGAGTCAACA	GCATCTGCTCAAAGTGTTCA	AATCAGAATGTCAGCTTGGC
GGTGACATTGGACACTCGAA	TGGTAAGATTGTCCTTGTCG	TATTGAGCTGTCGTTGCGTA
GACACTGAAGAGACGCTGTC	TATAGCCCATGCATCTGAAC	AGCACTGTTTCTCCACGAAA
GCTCTTGAGTAGATATCCTG	GTCAGATTGTCCTTGCAAAT	CAGACGGCTTGAATGAACCA
CACGCGATCGAGAACGAGAT	TCTCGAATTGTCAGGAGCAT	TTTGCGTGTGTCGAGAACAC
TGTGTCGATCCGATGAATGA	GTGTGATTCGTGTTTGGCAA	AGTCAGCAGCTCAACAACAT
GGAAATGCGTTGACTGGAGG	TCACGAGTATCGCAGAATCT	AAGCGACCTTCTTATTGACT
AAGAGTTGGCGGATGAAGGT	TAATCACATTCTCTTCTCCG	ATCCCTGTGATGTCTGAAAT
AGAGAAGAGCATCCGGTGAA	CTCGTGTGTACTAATCGGTA	TCTCTGGCATCTGTTTCAAA
GCGATTACGACTTGAACTGT	CCGCGTTGAGAAGATCATAT	TCCGGCCAAAAGTAGAACTT

Development • Supplementary information

References

Bachorik JL, Kimble J. 2005. Redundant control of the *Caenorhabditis elegans* sperm/oocyte switch by PUF-8 and FBF-1, two distinct PUF RNA-binding proteins. *Proc Natl Acad Sci U S A* **102**: 10893-10897.

Brenner S. 1974. The genetics of *Caenorhabditis elegans*. *Genetics* **77**: 71-94.

Crittenden SL, Bernstein DS, Bachorik JL, Thompson BE, Gallegos M, Petcherski AG, Moulder G, Barstead R, Wickens M, Kimble J. 2002. A conserved RNA-binding protein controls germline stem cells in *Caenorhabditis elegans*. *Nature* **417**: 660-663.

Ellis HM, Horvitz HR. 1986. Genetic control of programmed cell death in the nematode *C. elegans*. *Cell* **44**: 817-829.

Hengartner MO, Ellis RE, Horvitz HR. 1992. *Caenorhabditis elegans* gene *ced-9* protects cells from programmed cell death. *Nature* **356**: 494-499.

Nolde MJ, Saka N, Reinert KL, Slack FJ. 2007. The *Caenorhabditis elegans* pumilio homolog, *puf-9*, is required for the 3'UTR-mediated repression of the *let-7* microRNA target gene, *hbl-1*. *Dev Biol* **305**: 551-563.

Sarov M, Schneider S, Pozniakovski A, Roguev A, Ernst S, Zhang Y, Hyman AA, Stewart AF. 2006. A recombineering pipeline for functional genomics applied to *Caenorhabditis elegans*. *Nat Methods* **3**: 839-844.

Shaham S, Reddien PW, Davies B, Horvitz HR. 1999. Mutational analysis of the *Caenorhabditis elegans* cell-death gene *ced-3*. *Genetics* **153**: 1655-1671.

Subramaniam K, Seydoux G. 2003. Dedifferentiation of primary spermatocytes into germ cell tumors in *C. elegans* lacking the pumilio-like protein PUF-8. *Curr Biol* **13**: 134-139.

Zhong M, Niu W, Lu ZJ, Sarov M, Murray JI, Janette J, Raha D, Sheaffer KL, Lam HY, Preston E et al. 2010. Genome-wide identification of binding sites defines distinct functions for *Caenorhabditis elegans* PHA-4/FOXA in development and environmental response. *PLoS Genet* **6**: e1000848.

Development • Supplementary information
