# Supplementary material for: PUF-8, a C. elegans ortholog of the RNA-binding proteins PUM1 and PUM2, is required for robustness of the cell death fate
Source: Development. 2023 Oct 6;150(19):dev201167. doi: 10.1242/dev.201167 (PMC10565243; doi:10.1242/dev.201167)
Supplement: Supplementary file 1 [file develop-150-201167_review_history.pdf]

PUF-8, a *C. elegans* ortholog of the RNA-binding proteins PUM1 and PUM2, is required for robustness of the cell death fate

Jimei Xu, Yanwen Jiang, Ryan Sherrard, Kyoko Ikegami and Barbara Conradt
DOI: 10.1242/dev.201167

Editor: Swathi Arur

Review timeline
Original submission: 29 July 2022
Editorial decision: 5 September 2022
First revision received: 23 June 2023
Editorial decision: 26 July 2023
Second revision received: 8 September 2023
Accepted: 11 September 2023

Original submission

First decision letter

MS ID#: DEVELOP/2022/201167

MS TITLE: The C. elegans PUM1, 2-like RNA binding protein PUF-8 is required for robustness of the cell death fate

AUTHORS: Jimei Xu, Ryan Sherrard, Kyoko Ikegami, Ed E Large, Michael J Smanski, and Barbara Conradt

I have now received all the referees' reports on the above manuscript, and have reached a decision. As you will see, the referees express considerable interest in your work, but the study requires experimental evidence to show that PUF-8 directly binds to the 3'UTR of ced-3 and ced-9 and further mutate the binding residues to preclude the binding in vivo and test the function. I agree with the reviewers suggestions that this would be a critical experiment to conduct. In addition, each reviewer offers significant suggestions to improve the study that may require either experimental analysis or textual edits. If you are able to revise the manuscript along the lines suggested, I will be happy receive a revised version of the manuscript. Your revised paper will be re-reviewed by one or more of the original referees.

Please attend to all of the reviewers' comments and ensure that you clearly highlight all changes made in the revised manuscript. Please avoid using 'Tracked changes' in Word files as these are lost in PDF conversion. I should be grateful if you would also provide a point-by-point response detailing how you have dealt with the points raised by the reviewers in the 'Response to Reviewers' box. If you do not agree with any of their criticisms or suggestions please explain clearly why this is so.

Reviewer 1

Advance summary and potential significance to field

This manuscript describes a role for the RNA-binding protein PUF-8 in the regulation of developmentally controlled apoptosis in the RID lineage of the nematode worm C. elegans. The authors use genetics methods to show that PUF-8 prevents inappropriate death of cells by

© 2023. Published by The Company of Biologists under the terms of the Creative Commons Attribution License (https://creativecommons.org/licenses/by/4.0/).

repressing the caspase ced-3. They also propose that PUF-8 has pro-apoptotic activity through the repression of the anti-apoptotic gene ced-9. Previously, PUF-8 was shown to repress ced-3 in the mitotic region of the C. elegans germline (Subasic et al., 2015) but no evidence that it affects ced-9 has been published before this study. The authors propose a model whereby PUF-8 serves to fine-tune the regulation of apoptosis through its pro- and anti-apoptotic activities on ced-3 and ced-9. This work provides new insights into how survival of cells in the RID lineage is regulated, which has broader implications of cell death regulatory mechanisms across phyla.

Comments for the author

Overall, the only issue I have with this work is how PUF-8 both promotes and represses apoptosis. To help clarify this issue the authors should perform one of the following two experiments.

1. Does PUF-8 bind to the 3'UTR of ced-3 and ced-9 with different affinities? This could be done by a simple in vitro binding assay with recombinant protein and RNA to each gene.
2. What is the consequence of mutating the PUF-8 binding sites in the ced-3 and ced-9 genes? Does this affect apoptosis of RID lineage in a wild-type (or sensitized) background?

Minor comment
Pg 10, second paragraph, second line: n242 should be n2427.

Reviewer 2

Advance summary and potential significance to field

The manuscript by Xu et al, describes the fine tuning of C. elegans apoptosis regulation by PUF-8, a C. elegans PUM1, 2 like mRNA binding protein known that act by regulating mRNA turnover, and or translation. Taking advantage of C. elegans developmental lineaging, and the fact that C elegans embryonic development is largely invariant, 131 cells normally succumbing to apoptosis the authors show cases where in puf-8 mutants excessive and/or early apoptosis occurs in some lineages, while apoptosis is reduced in other linages, examples of both happing in the same lineage also being shown. Excessive (or precautious) apoptosis occurs during all three embryonic waves of developmental apoptosis. The phenotypes, are generally weak, but nevertheless highly convincing developmental lineaging allowing to precisely quantify individual developmental apoptosis events. The standard of these experiments is high, all controls such as ascertaining a phenotype by a second puf-8 allele, and complementation are done. Finding excessive apoptosis is further bolstered by showing synthetic lethality with a temperature sensitive ced-9 allele, the lethality being blocked by mutating the C. elegans ced-3 downstream apoptosis gene. The nematode provides a unique system to conduct such previse analysis of apoptosis which is impossible in any other system.

Focusing, in on individual death events, mindful that PUF-1 mRNA binding sites are predicted to occur in 3'UTRs of ced-3, and ced-4 proapoptotic genes, and the ced-9 apoptosis protector the authors measure mRNA levels (by direct in single molecule in situ hybridization) and protein levels using reporters. In the ring neuroblast lineage (RID), tallying cases of precocious apoptosis phenotype, ced-4 and ced-3 expression is increased in the mother cells that normally does not die, while in line with cases of daughter cells that normally die, the mRNA expression of ced-9 mRNA appears to be increased (when these cells do not die). I think that the quantifications based on single molecule mRNA situ hybridization experiments, which provide quantal information, (Figure 4) are valid, however I am not so sure about the quantification shown in Figure 4 (ced-3 transcriptional reporter), which I understand describes the level of GFP, that replaces the CED-3 coding region. GFP appears to be localized in granular structures and I am not sure how valid it is to measure mean fluorescent intensity in such a situation, all the more so that differences are not too big. Would it not be better to fuse the GFP to a nuclear localization signal this way much better being able to measure the intensity of locally confined GFF? Also, taking histone variant Cherry fusion could/ should be taken as internal control. The critique relating to quantification also applies for Figure 5 (CED-9 translational reporter). Of course in this case reliable quantification is even more difficult, focusing the CED-9 protein to a distinct subcellular localization for

© 2023. Published by The Company of Biologists under the terms of the Creative Commons Attribution License (https://creativecommons.org/licenses/by/4.0/).

quantification not being a valid approach. Use of internal control? All in all, I acknowledge that mRNA and especially protein quantification is probably most challenging in this experimental system. Again single transcript measurements appear appropriate. Transcriptional reporters can/ should be improved. CED-9 protein quantification might well be a 'bridge to far'.

Major weaknesses.

As stated above I am not entirely convinced about the quantification provided in Figure 4 (ced-3 transcriptional reporter) which to my mind could be improved in principle) and Figure 5 (ced-9 translational reporter) which may well not be doable.

At present, and to the defense the authors acknowledge this in their discussion the authors do not show if PUF-8 directly binds to 'PUF' binding elements they found in 3' UTRs of ced-9, ced-4 and ced-3 apoptosis genes. Also, they do not replace the respective 3'UTRs, with a 'constitutive 3'-UTR, or mutate individual binding elements and assess if this affects mRNA turnover, or the expression level of the corresponding proteins. These experiments, with the advance of genome editing, are doable. To my mind it is an editorial decision of the editors of Development to decide if this study is sufficiently 'mechanistic' to warrant publication, the genetics is of high quality for sure. Would it make sense to report if PUF binding elements are enriched in the 3'UTRs of apoptosis genes, compared to a random set of genes?

All in all, the data described in this paper is interesting. The quality of the genetic data, and to my mind also the quality of single mRNA quantifications is high. Yes, I agree, that evidence is provided that PUF-8 might help to maintain the precision of transcriptional regulation. I am not convinced, about the quantifications shown in Figure 4 and 5. I think there is a way to improve the analysis of transcriptional reporters. For, the analysis of the CED-9 translational reporter I am not sure. Would it make sense to tune down this finding, and maybe show changes in transcription levels of apoptosis genes in a second sub lineage focusing the more assessing transcription? Alternative replacement of 3'UTR as a functional output, might yield results more straight forward compared to measuring the level of apoptosis proteins.

Minor comments:

I think the authors, at the end of the introduction or the beginning of the results section might want to better explain why they specifically started to look at the effects of puf-8 deletion. Was this a result of some initial screening through known or suspected mRNA binding protein?

C. elegans PUF-8 and PUF-9 are described as the 'orthologs' of mammalian PUM1 and PUM2. The authors in a half sentence might want to better describe the phylogenetic relationship of this proteins. Are PUF-8 and PUF-9 and PUM1 and PUM2 derived from separate linage specific duplications (eg nematodes, and vertebrates), or did the duplication already occur before the separation of nematodes and vertebrates.

The authors show that the lethality of puf-8 ced-9 double mutants is largely blocked by ced-3. Is the low-level lethality of puf-8 also blocked by ced-3 mutation?

End of introduction section. 'In addition, our results…..' I think the authors should reword 'a little' saying that their new results provide a further/additional mechanism for regulating the developmental robustness of cell fate decision in addition to the previously described role of micro-RNAi mediated regulation. As is they authors imply that they have analyzed the combined effect of micro-RNAi and the PUF RNA binding protein in this manuscript, which they have not done.

Page 13. Title. Impact of loss of puf-8……

Reword to make it clearer that you now looked at the ced-3 mRNA expression using ced-3 promoter fusion (mjs7) where the ced-3 open reading frame is replaced by GFP

Page 14
Title, change 'ced-9' to 'CED-9' to indicate upfront that this is a translational fusion.

© 2023. Published by The Company of Biologists under the terms of the Creative Commons Attribution License (https://creativecommons.org/licenses/by/4.0/).

Comments for the author

see above

Reviewer 3

Advance summary and potential significance to field

Summary:
The manuscript by Xu et al report that the conserved RNA-binding protein PUF-8 has both pro- and anti-apoptotic function during C. elegans embryogenesis. They find that a small percentage of cells die through the apoptotic pathway in the puf-8 mutant animals. They also find that a small percentage of cells that are normally fated to die survive or take longer to die in puf-8 animals. Furthermore, they find a slight, but statistically significant, increase in the mRNA levels of ced-3, which encodes a protease that is central to apoptosis, in the ring interneuron D neuroblast (RIDnb) in the puf-8 mutant embryos; RIDnb is one of the cells that precociously die in some of the puf-8 mutant embryos. They also find a slight but statistically significant increase in ced-9 mRNA levels in RIDsc, the daughter of RIDnb that is normally fated to die but survives in a few puf-8 mutant embryos. Consistently, the expression of the GFP reporter under the control of ced-3 and ced-9 3' UTRs also increase in the absence of PUF-8. These results suggest that the observed PUF-8's anti- and pro-apoptotic activities are due to its influence on the stability of ced-3 and ced-9 mRNAs.

Potential significance:
Results reported here, assuming that the authors will be able to establish that the PUF-8's anti-and pro-apoptotic functions are through its direct influence on the levels of ced-3 and ced-9 mRNA levels, show how the PUF proteins balance between opposite decisions by fine-tuning the expression of genes at the posttranscriptional level and bring robustness to the decisions made. This is consistent with the earlier results on PUF-8' function in the germ line.

Comments for the author

The current observations are correlations, and do not demonstrate cause-effect relationship between PUF-8 and ced-3/ced9. Since only a small percentage of cells die in the puf-8 mutant, one can't be sure, for example, if ced-3 mRNA level increased in the RIDnb that precociously died in the puf-8 mutant. Similar argument holds good for the observed pro-apoptotic activity as well. In addition, the mechanisms that underlie why PUF-8 acts on the stability of ced-3 and ced-9 mRNAs only in RIDnb and RIDsc, respectively, but on in RID, the other daughter of RIDnb, is not clear. One way to demonstrate the cause-effect relationship is to test if mutations of the potential PUF-8-binding elements in the ced-3 and ced-9 3' UTRs at the endogenous loci cause cell-death phenotypes similar to the ones observed in the puf-8 mutant animals.

My other comments are listed below:
Page 9, last line – There is a considerable difference between q725 and ok302. Is this significant? If yes, it will be good to comment / explain why such a difference between the two null alleles.

Page 10, second paragraph, last sentence – If this conclusion is true, then most, if not all, cells programmed to die should survive in animals homozygous for the puf-8 null alleles?

Page 12, second paragraph, third sentence - The reported values are average of how many cells for all three cell types? I see that the individual values have been presented as dots in the graph, but it is not easy to count them; will be easier if the "n" value is mentioned.

Page 13, second paragraph, fourth sentence – there is a concern in using the mjs7 allele: absence of the encoded protein may influence the expression of the gene. Since this is one of the key data, it will important to rule out this possibility.

Page 14, third line from the top – include representative images of the puf-8 mutant embryos in Figure S4. The figure number is cited wrongly, it should have been 4 instead of 5.

© 2023. Published by The Company of Biologists under the terms of the Creative Commons Attribution License (https://creativecommons.org/licenses/by/4.0/).

Page 15, the concluding sentence - If slight increase in CED-9 levels could protect RIDsc from cell death, one would expect to see some cells that are fated to die to survive in lines expressing the Pced-9::GFP::CED-9 transgene. Was this observed?

Page 37 – Legend and figure labels have been swapped between D and E in Figure 1.

Page 38 – Legend and figure labels have been swapped between D and E in Figure 2.

Page 38 – What is the value of the scale bars in Figure 3B?

First revision

Author response to reviewers' comments

Reviewer 1

Advance Summary and Potential Significance to Field:
This manuscript describes a role for the RNA-binding protein PUF-8 in the regulation of developmentally controlled apoptosis in the RID lineage of the nematode worm C. elegans. The authors use genetics methods to show that PUF-8 prevents inappropriate death of cells by repressing the caspase ced-3. They also propose that PUF-8 has pro-apoptotic activity through the repression of the anti-apoptotic gene ced-9. Previously, PUF-8 was shown to repress ced-3 in the mitotic region of the C. elegans germline (Subasic et al., 2015) but no evidence that it affects ced-9 has been published before this study. The authors propose a model whereby PUF-8 serves to fine-tune the regulation of apoptosis through its pro- and anti-apoptotic activities on ced-3 and ced-9. This work provides new insights into survival of cells in the RID lineage is regulated, which has broader implications of cell death regulatory mechanisms across phyla.

Comments for the Author:
Overall, the only issue I have with this work is how PUF-8 both promotes and represses apoptosis. To help clarify this issue the authors should perform one of the following two experiments.

1. Does PUF-8 bind to the 3'UTR of ced-3 and ced-9 with different affinities?
This could be done by a simple in vitro binding assay with recombinant protein and RNA to each gene.

Xu et al
In response to all three Reviewers' comments, we have performed PUF-8 pull down experiments. Specifically, we used a puf-8 CRISPR allele that generates 3xFLAG-tagged PUF-8 protein from the endogenous locus, pulled down 3xFLAG::PUF-8 protein from worm lysates using a FLAG antibody and tested the protein pulled down for the presence of egl-1, ced-9, ced-4 or ced-3 mRNAs. Using this approach, we found that ced-3 mRNA is significantly enriched over the control (pulled down ATFS-1::EGFP::3xFLAG protein), ced-9 and ced-4 mRNAs are enriched but not in a statistically significant manner and essentially no enrichment is seen for egl-1 mRNA (see new Figure 4). Based on this we propose that PUF-8 directly binds to ced-3 mRNA and that it may also directly bind to ced-9 and ced-4 mRNAs. These results also suggest that there is a difference in the affinity of PUF-8 for ced-3 and ced- 9 mRNAs.

2. What is the consequence of mutating the PUF-8 binding sites in the ced-3 and ced-9 genes? Does this affect apoptosis of RID lineage in a wild-type (or sensitized) background?

Xu et al
Since the results we obtained from the pull-down experiments demonstrate that PUF-8 protein physically interacts with ced-3 mRNAs, we performed the experiment suggested by the Reviewer for the ced-3 gene. Specifically, we mutated all 6 PUF-binding elements (PBEs) in the ced-3 3'UTR

© 2023. Published by The Company of Biologists under the terms of the Creative Commons Attribution License (https://creativecommons.org/licenses/by/4.0/).

using CRISPR (ced-3 allele bc448) and analysed ced-3(bc448) for a cell death phenotype. We found that ced-3(bc448) by itself does not cause ectopic or precocious death ('large corpses' phenotype) and it also does not cause embryonic lethality. However, ced-3(bc448) significantly enhances the large cell corpse phenotype caused by the ced-9 loss- of-function allele n1653ts at 25°C (from 4.8 large corpses/embryo to 10.1 large corpses/embryo; see new Figure 5). In addition, ced-3(bc448) significantly increases embryonic lethality in ced-9(n1653ts) animals from 13.9 to 45.5 % at 20°C. This is lower than the embryonic lethality observed in puf-8(q725); ced-9(n1653ts) animals, suggesting that puf-8 may have targets other than ced-3 mRNA that contribute to this lethality (ced-4 mRNA?). However, introducing ced-3(bc448) into puf-8(q725); ced-9(n1653ts) does not increase lethality indicating that the impact of ced-3(bc448) on embryonic lethality in the ced-9(n1653ts) background is mediated by PUF-8 and not other RBPs that may bind to the six PBEs. In summary, these results confirm our model that PUF-8 protein directly binds to PBEs in the 3'UTR of ced-3 mRNAs thereby preventing ectopic and precocious death (most probably by reducing ced-3 expression). In addition, ced-3(bc448) is unlike loss-of-function mutations of ced-3 which suppress rather than enhance cell death and can be considered a weak 'gain-of-function' mutation.

Minor comment
Pg 10, second paragraph, second line: n242 should be n2427.

Xu et al
Fixed.

Reviewer 2
Advance Summary and Potential Significance to Field:
The manuscript by Xu et al, describes the fine tuning of C. elegans apoptosis regulation by PUF-8, a C. elegans PUM1, 2 like mRNA binding protein known that act by regulating mRNA turnover, and or translation. Taking advantage of C. elegans developmental lineaging, and the fact that C elegans embryonic development is largely invariant, 131 cells normally succumbing to apoptosis, the authors show cases where in puf-8 mutants excessive and/or early apoptosis occurs in some lineages, while apoptosis is reduced in other linages, examples of both happing in the same lineage also being shown. Excessive (or precautious) apoptosis occurs during all three embryonic waves of developmental apoptosis. The phenotypes, are generally weak, but nevertheless highly convincing, developmental lineaging allowing to precisely quantify individual developmental apoptosis events. The standard of these experiments is high, all controls such as ascertaining a phenotype by a second puf-8 allele, and complementation are done. Finding excessive apoptosis is further bolstered by showing synthetic lethality with a temperature sensitive ced-9 allele, the lethality being blocked by mutating the C. elegans ced-3 downstream apoptosis gene. The nematode provides a unique system to conduct such previse analysis of apoptosis which is impossible in any other system.

Comments for the Author:
Focusing, in on individual death events, mindful that PUF-1 mRNA binding sites are predicted to occur in 3'UTRs of ced-3, and ced-4 proapoptotic genes, and the ced-9 apoptosis protector the authors measure mRNA levels (by direct in single molecule in situ hybridization) and protein levels using reporters. In the ring neuroblast lineage (RID), tallying cases of precocious apoptosis phenotype, ced-4 and ced-3 expression is increased in the mother cells that normally does not die, while in line with cases of daughter cells that normally die, the mRNA expression of ced-9 mRNA appears to be increased (when these cells do not die). I think that the quantifications based on single molecule mRNA situ hybridization experiments, which provide quantal information, (Figure 4) are valid, however I am not so sure about the quantification shown in Figure 4 (ced-3 transcriptional reporter), which I understand describes the level of GFP, that replaces the CED-3 coding region. GFP appears to be localized in granular structures and I am not sure how valid it is to measure mean fluorescent intensity in such a situation, all the more so that differences are not too big. Would it not be better to fuse the GFP to a nuclear localization signal this way much better being able to measure the intensity of locally confined GFF? Also, taking histone variant Cherry fusion could/ should be taken as internal control.

The critique relating to quantification also applies for Figure 5 (CED-9 translational reporter). Of course in this case reliable quantification is even more difficult, focusing the CED-9 protein to a

© 2023. Published by The Company of Biologists under the terms of the Creative Commons Attribution License (https://creativecommons.org/licenses/by/4.0/).

distinct subcellular localization for quantification not being a valid approach. Use of internal control? All in all, I acknowledge that mRNA and especially protein quantification is probably most challenging in this experimental system. Again, single transcript measurements appear appropriate. Transcriptional reporters can/ should be improved. CED-9 protein quantification might well be a ‘bridge to far’.

Xu et al
We appreciate that the Reviewer considers the quantification of the single molecule RNA FISH data valid (Figure 3). And we agree with the Reviewer that protein quantification in single cells in developing embryos is challenging (and possibly impossible at the moment), especially for proteins such as CED-9, which localises to mitochondria. For this reason, we decided to remove Figure 5. In addition, we agree that the ced-3 reporter (which was not a purely transcriptional reporter since it contained the endogenous ced-3 3’ UTR) was not ideal but we are concerned about artificially targeting endogenous CED-3 protein into the nucleus. Therefore, we decided to also remove Figure 4. Finally, we decided not to generate improved transcriptional reporters, since puf-8 exerts its effect on gene expression at the post- transcriptional level.

Major weaknesses.

As stated above I am not entirely convinced about the quantification provided in Figure 4 (ced-3 transcriptional reporter) which to my mind could be improved in principle) and Figure 5 (ced-9 translational reporter) which may well not be doable.

Xu et al
As mentioned above, in response to Reviewer 2’s comments, we have removed these two figures.

At present, and to the defense the authors acknowledge this in their discussion, the authors do not show if PUF-8 directly binds to ‘PUF’ binding elements they found in 3’ UTRs of ced-9, ced-4 and ced-3 apoptosis genes. Also, they do not replace the respective 3’UTRs, with a ‘constitutive 3’-UTR, or mutate individual binding elements and assess if this affects mRNA turnover, or the expression level of the corresponding proteins. These experiments, with the advance of genome editing, are doable. To my mind it is an editorial decision of the editors of Development to decide if this study is sufficiently ‘mechanistic’ to warrant publication, the genetics is of high quality for sure. Would it make sense to report if PUF binding elements are enriched in the 3’UTRs of apoptosis genes, compared to a random set of genes?

All in all, the data described in this paper is interesting. The quality of the genetic data, and to my mind also the quality of single mRNA quantifications is high. Yes, I agree, that evidence is provided that PUF-8 might help to maintain the precision of transcriptional regulation. I am not convinced, about the quantifications shown in Figure 4 and 5. I think there is a way to improve the analysis of transcriptional reporters. For, the analysis of the CED-9 translational reporter I am not sure. Would it make sense to tune down this finding, and maybe show changes in transcription levels of apoptosis genes in a second sub lineage focusing the more assessing transcription? Alternative, replacement of 3’UTR as a functional output, might yield results more straight forward compared to measuring the level of apoptosis proteins.

Xu et al
As mentioned above, in response to Reviewer 2’s comments, Figures 4 and 5 have been removed from the revised manuscript. Also as mentioned above, since puf-8 exerts its impact on gene expression at the post-transcriptional level, we believe that looking at change in the level of transcription of apoptosis genes will not be informative.

However, as outlined in our response to Reviewer 1’s comments and as outlined again below, we have performed additional experiments (PUF-8 pull-down, genome editing of PBEs; see new Figures 4 and 5) that provide evidence that PUF-8 protein physically interacts with ced-3 mRNA (and possibly also with ced-4 and ced-9 mRNAs) and that mutating the PBEs in the 3’ UTR of ced-3 mRNA has an impact on cell death and on puf-8’s ability to impact cell death.

In response to the Reviewers’ comments, we have performed PUF-8 pull down experiments. Specifically, we used a puf-8 CRISPR allele that generates 3xFLAG-tagged PUF-8 protein from the

© 2023. Published by The Company of Biologists under the terms of the Creative Commons Attribution License (https://creativecommons.org/licenses/by/4.0/).

endogenous locus, pulled down 3xFLAG::PUF-8 protein from worm lysates using a FLAG antibody and tested the protein pulled down for the presence of egl-1, ced-9, ced-4 or ced-3 mRNAs. Using this approach, we found that ced-3 mRNA is significantly enriched over the control (pulled down ATFS-1::EGFP::3xFLAG protein), ced-9 and ced-4 mRNAs are enriched but not in a statistically significant manner and essentially no enrichment is seen for egl-1 mRNA (see new Figure 4). Based on this we propose that PUF-8 directly binds to ced-3 mRNA and that it may also directly bind to ced-9 and ced-4 mRNAs.

Since the results we obtained from the pull-down experiments demonstrate that PUF-8 protein physically interacts with ced-3 mRNAs, we mutated all 6 PUF-binding elements (PBEs) in the ced-3 3'UTR using CRISPR (ced-3 allele bc448) and analysed ced-3(bc448) for a cell death phenotype. We found that ced-3(bc448) by itself does not cause ectopic or precocious death ('large corpses' phenotype) and it also does not cause embryonic lethality. However, ced-3(bc448) significantly enhances the large cell corpse phenotype caused by the ced-9 loss-of-function allele n1653ts at 25°C (from 4.8 large corpses/embryo to 10.1 large corpses/embryo; see new Figure 5). In addition, ced-3(bc448) significantly increases embryonic lethality in ced-9(n1653ts) animals from 13.9 to 45.5 % at 20°C. This is lower than the embryonic lethality observed in puf-8(q725); ced-9(n1653ts) animals, suggesting that puf-8 may have targets other than ced-3 mRNA that contribute to this lethality (ced-4 mRNA?). However, introducing ced-3(bc448) into puf-8(q725); ced-9(n1653ts) does not increase lethality indicating that the impact of ced-3(bc448) on embryonic lethality in the ced-9(n1653ts) background is mediated by PUF-8 and not other RBPs that may bind to the six PBEs. In summary, these results confirm our model that PUF-8 protein directly binds to PBEs in the 3'UTR of ced-3 mRNAs thereby preventing ectopic and precocious death (most probably reducing ced-3 expression). In addition, ced-3(bc448) is unlike loss-of-function mutations of ced-3 (which suppress rather than enhance cell death) and can be considered a weak 'gain-of-function' mutation.

Minor comments:

I think the authors, at the end of the introduction or the beginning of the results section might want to better explain why they specifically started to look at the effects of puf-8 deletion. Was this a result of some initial screening through known or suspected mRNA binding protein?

Xu et al
To address the Reviewer's comment, we have changed the third sentence of the Result section from:

"To determine whether puf-8$^{PUM1, 2}$ has roles in somatic tissues during embryogenesis, using a four-dimensional (4D) microscope system (Schnabel et al. 1997), we followed embryonic development of wild-type C. elegans embryos and embryos homozygous for q725 or ok302, two strong loss-of-function (lf) mutations of puf-8 (Subramaniam and Seydoux 2003; Bachorik and Kimble 2005)."

to:
"As part of a small scale screen for RBP genes with a role in developmental robustness, using a four-dimensional (4D) microscope system (Schnabel et al. 1997), we followed embryonic development of embryos homozygous for q725 or ok302, two strong loss-of- function (lf) mutations of puf-8 (Subramaniam and Seydoux 2003; Bachorik and Kimble 2005)."

C. elegans PUF-8 and PUF-9 are described as the 'orthologs' of mammalian PUM1 and PUM2. The authors in a half sentence might want to better describe the phylogenetic relationship of this proteins. Are PUF-8 and PUF-9 and PUM1 and PUM2 derived from separate linage specific duplications (eg nematodes, and vertebrates), or did the duplication already occur before the separation of nematodes and vertebrates.

Xu et al
To address the Reviewer's comment, we have added the following sentence:

"(The PUM1/2 and PUF-8/9 duplication is likely to have occurred before the separation of nematodes and vertebrates (Spassov and Jurecic 2003).)"

© 2023. Published by The Company of Biologists under the terms of the Creative Commons Attribution License (https://creativecommons.org/licenses/by/4.0/).

The authors show that the lethality of puf-8 ced-9 double mutants is largely blocked by ced-3. Is the low-level lethality of puf-8 also blocked by ced-3 mutation?

Xu et al
As shown in Figure 1E, the low level of lethality in puf-8(q725) animals (5.8%) is not blocked by the loss of ced-3; indeed, it seems to be enhanced (17.1%). We currently do not have an explanation for this but suggest that this enhancement is the result of puf-8 ced-3 interactions that are independent of apoptotic cell death.

End of introduction section. ‘In addition, our results…..’ I think the authors should reword ‘a little’ saying that their new results provide a further/additional mechanism for regulating the developmental robustness of cell fate decision in addition to the previously described role of micro-RNAi mediated regulation. As is they authors imply that they have analyzed the combined effect of micro-RNAi and the PUF RNA binding protein in this manuscript, which they have not done.

Xu et al
The Reviewer is referring to the last sentence of the introduction:

“In addition, our results indicate that in the context of the C. elegans cell death fate, the roles in developmental robustness of microRNAs and RBPs are non-overlapping and, hence, complementary.”

In response to the Reviewer’s comment, we have deleted this sentence.

Page 13. Title. Impact of loss of puf-8……

Reword to make it clearer that you now looked at the ced-3 mRNA expression using ced-3 promoter fusion (mjs7) where the ced-3 open reading frame is replaced by GFP

Xu et al
This paragraph has been removed because Figure 4 was removed.

Page 14
Title, change ‘ced-9’ to ‘CED-9’ to indicate upfront that this is a translational fusion.

Xu et al
This paragraph has been removed because Figure 5 was removed.

Reviewer 3
Advance Summary and Potential Significance to Field: Summary:
The manuscript by Xu et al report that the conserved RNA-binding protein PUF-8 has both pro- and anti-apoptotic function during C. elegans embryogenesis. They find that a small percentage of cells die through the apoptotic pathway in the puf-8 mutant animals. They also find that a small percentage of cells that are normally fated to die survive or take longer to die in puf-8 animals. Furthermore, they find a slight, but statistically significant, increase in the mRNA levels of ced-3, which encodes a protease that is central to apoptosis, in the ring interneuron D neuroblast (RIDnb) in the puf-8 mutant embryos; RIDnb is one of the cells that precociously die in some of the puf-8 mutant embryos. They also find a slight but statistically significant increase in ced-9 mRNA levels in RIDsc, the daughter of RIDnb that is normally fated to die but survives in a few puf-8 mutant embryos. Consistently, the expression of the GFP reporter under the control of ced-3 and ced-9 3’ UTRs also increase in the absence of PUF-8. These results suggest that the observed PUF-8’s anti- and pro-apoptotic activities are due to its influence on the stability of ced-3 and ced-9 mRNAs.

Potential significance:
Results reported here, assuming that the authors will be able to establish that the PUF-8’s anti-and pro-apoptotic functions are through its direct influence on the levels of ced-3 and ced-9 mRNA levels, show how the PUF proteins balance between opposite decisions by fine- tuning the expression of genes at the posttranscriptional level and bring robustness to the decisions made.

© 2023. Published by The Company of Biologists under the terms of the Creative Commons Attribution License (https://creativecommons.org/licenses/by/4.0/).

This is consistent with the earlier results on PUF-8' function in the germ line.
Comments for the Author:
The current observations are correlations, and do not demonstrate cause-effect relationship between PUF-8 and ced-3/ced9. Since only a small percentage of cells die in the puf-8 mutant, one can't be sure, for example, if ced-3 mRNA level increased in the RIDnb that precociously died in the puf-8 mutant. Similar argument holds good for the observed pro- apoptotic activity as well. In addition, the mechanisms that underlie why PUF-8 acts on the stability of ced-3 and ced-9 mRNAs only in RIDnb and RIDsc, respectively, but on in RID, the other daughter of RIDnb, is not clear. One way to demonstrate the cause-effect relationship is to test if mutations of the potential PUF-8-binding elements in the ced-3 and ced-9 3' UTRs at the endogenous loci cause cell-death phenotypes similar to the ones observed in the puf-8 mutant animals.

Xu et al

We agree with the Reviewer that an ideal experiment would be to measure for example ced-3 mRNA levels in single RID neuroblasts in puf-8 mutants and to then follow the fate of these cells. However, for technical reasons and because of the low penetrance of the puf-8 phenotype, we are currently unable to perform this experiment.

However, as described in our responses to Reviewer 1 and 2's comments, we have now obtained evidence that PUF-8 directly interacts with ced-3 mRNA in vivo. In addition, we have generated, through CRISPR/Cas, a ced-3 allele in which the 6 PBEs in the ced-3 3' UTR are mutated. The resulting ced-3(bc448) allele enhances the large cell corpse phenotype and the Emb phenotype of ced-9(n1653ts) animals and hence, has a phenotype weaker than but similar to that of animals lacking puf-8. (We consider it a possibility that PUF-8 also targets ced-4, which would explain why the ced-3(bc448) phenotype is weaker than that of the loss of puf-8.)

My other comments are listed below:
Page 9, last line – There is a considerable difference between q725 and ok302. Is this significant? If yes, it will be good to comment / explain why such a difference between the two null alleles.

Xu et al
In response to the Reviewer's comment regarding the data shown in Figure 2B, we have done a Mann-Whitney test and found that the P-value between the puf-8 alleles q725 and ok302 is 0.0005 (now labelled with 2 stars in Figure 2B). Since q725 and ok302 differ only with respect to the loss of puf-8's pro-apoptotic activity and since q725 is fully rescued by a puf-8(+) transgene, we propose that this difference is caused by a mutation in the background of ok302, which is the stronger allele in this context. We have added the following sentence at the end of page 9 of the manuscript:

"(Of note, the difference between puf-8(q725) and puf-8(ok302) is statistically significant and may be caused by a mutation in the puf-8(ok302) background.) "

Page 10, second paragraph, last sentence – If this conclusion is true, then most, if not all, cells programmed to die should survive in animals homozygous for the puf-8 null alleles?

Xu et al
The Reviewer is referring to the following sentence:

"This pro-apoptotic activity ensures that cells programmed to die reproducibly adopt the cell death fate and that apoptotic cell death is swiftly executed."

What we meant to convey is that in the absence of puf-8 these cells no longer **reproducibly** die but instead, sometimes survive. For example, in puf-8 mutants, the RIDsc survives 3.2% of the times. We have changed the sentence and hope that this is no longer misleading:

"This pro-apoptotic activity ensures that these cells adopt the cell death fate 100% of the time and that the cell death fate is swiftly executed."

Page 12, second paragraph, third sentence - The reported values are average of how many cells for all three cell types? I see that the individual values have been presented as dots in the graph, but it

© 2023. Published by The Company of Biologists under the terms of the Creative Commons Attribution License (https://creativecommons.org/licenses/by/4.0/).

is not easy to count them; will be easier if the “n” value is mentioned.

Xu et al
In response to the Reviewer’s comment, we have added this information to the legend of Figure 3C. The n’s for the various cells and mRNAs are the following:

The number of cells for *egl-1* in RIDnb are n=10 for +/+, n=12 for *puf-8(q725)*;
The number of cells for *ced-9* in RIDnb are n=6 for +/+, n=12 for *puf-8(q725)*;
The number of cells for *ced-4* in RIDnb are n=9 for +/+, n=16 for *puf-8(q725)*;
The number of cells for *ced-3* in RIDnb are n=17 for +/+, n=34 for *puf-8(q725)*;

The number of cells for *egl-1* in RID are n=14 for +/+, n=15 for *puf-8(q725)*;
The number of cells for *ced-9* in RID are n=12 for +/+, n=12 for *puf-8(q725)*;
The number of cells for *ced-4* in RID are n=6 for +/+, n=14 for *puf-8(q725)*;
The number of cells for *ced-3* in RID are n=17 for +/+, n=14 for *puf-8(q725)*;

The number of cells for *egl-1* in RIDsc are n=14 for +/+, n=13 for *puf-8(q725)*;
The number of cells for *ced-9* in RIDsc are n=12 for +/+, n=12 for *puf-8(q725)*;
The number of cells for *ced-4* in RIDsc are n=6 for +/+, n=14 for *puf-8(q725)*;
The number of cells for *ced-3* in RIDsc are n=17 for +/+, n=14 for *puf-8(q725)*;

Page 13, second paragraph, fourth sentence – there is a concern in using the mjs7 allele: absence of the encoded protein may influence the expression of the gene. Since this is one of the key data, it will important to rule out this possibility.

Xu et al
As outlined in our response to Reviewer 2, Figures 4 and 5, including the data on mjs7, have been removed.

Page 14, third line from the top – include representative images of the puf-8 mutant embryos in Figure S4. The figure number is cited wrongly, it should have been 4 instead of 5.

Xu et al
Since Figures 4 and 5 were removed, we also removed Figure S4.

Page 15, the concluding sentence - If slight increase in CED-9 levels could protect RIDsc from cell death, one would expect to see some cells that are fated to die to survive in lines expressing the Pced-9::GFP::CED-9 transgene. Was this observed?

Xu et al
The data concerning Pced-9::GFP::CED-9::ced-9 3’ UTR (*bcSi130)* has been removed. We know that bcSi130 rescues the loss of ced-9, but we have not specifically looked for extra cells in strains carrying this allele.

Page 37 – Legend and figure labels have been swapped between D and E in Figure 1.

Xu et al
Fixed.

Page 38 – Legend and figure labels have been swapped between D and E in Figure 2.

Xu et al
Fixed.

Page 38 – What is the value of the scale bars in Figure 3B?
Xu et al
The scale bar in Figure 3B is 2 µm. In our revised manuscript, we have added ‘2 µm’ to the legend of Figure 3B.

© 2023. Published by The Company of Biologists under the terms of the Creative Commons Attribution License (https://creativecommons.org/licenses/by/4.0/).

Second decision letter

MS ID#: DEVELOP/2022/201167

MS TITLE: The C. elegans PUM1, 2-like RNA binding protein PUF-8 is required for robustness of the cell death fate

AUTHORS: Jimei Xu, Yanwen Jiang, Ryan Sherrard, Kyoko Ikegami, and Barbara Conradt

I have now received all the referees reports on the above manuscript, and have reached a decision. The referees' comments are appended below, or you can access them online: please go to BenchPressand click on the 'Manuscripts with Decisions' queue in the Author Area.

The overall evaluation is positive and we would like to publish a revised manuscript in Development. Reviewer 2 and 3 provide recommendations which will further improve the clarity of the paper, which can be addressed textually and will enhance the rigor of the analysis. I do not expect to send the manuscript back to the reviewers, however, I do ask that you please attend to all of the reviewers' comments in your revised manuscript and detail them in your point-by-point response, since I will review the submitted version. If you do not agree with any of their criticisms or suggestions explain clearly why this is so. If it would be helpful, you are welcome to contact us to discuss your revision in greater detail. Please send us a point-by-point response indicating your plans for addressing the referee's comments, and we will look over this and provide further guidance.

Reviewer 1

Advance summary and potential significance to field

This manuscript demonstrates a direct interaction of the PUF-8 protein with the 3'UTR of the ced-3 gene, which regulates developmentally controlled apoptosis in C. elegans. This is an important advance because it describes a new and important layer of regulation of programmed cell death. This sets the stage for other groups to investigate conserved roles of homologous RNA-binding proteins in vertebrates.

Comments for the author

I am satisfied with revised manuscript and recommend publication.

Reviewer 2

Advance summary and potential significance to field

see below

Comments for the author

The revised manuscript by Xu et al., is very much improved and to my mind warrants publication with minor revisions. I think that it was wise to remove Figures 4 and 5 focused on apoptosis protein expression as such measurements in subcompartments of single cells and organelles (mitochondria CED-9) in the context if an entire organism are most difficult/ next to impossible, especially given that differentials are not expected to be 'black and white'. The new evidence showing directed PUF-8 ced-3 mRNA binding, and the binding sites being required in vivo are all the more convincing. Congratulations!

© 2023. Published by The Company of Biologists under the terms of the Creative Commons Attribution License (https://creativecommons.org/licenses/by/4.0/).

Major Point
Sorry to have missed this earlier on: It is imperative to provide a full strain list using 'Conradt lab' designations. Providing this is most important for other labs to use the reagents and, if necessary, reproduce the data. Most of the alleles used in this study were still generated by classic genetic, whole genome mutagenesis-based methods where modifying mutations may remain, even after excessive backcrossing. Indeed, the authors imply that the differences observed between the strains carrying puf-8(q725) and puf-8 (ok302) alleles (both of which are likely to be null alleles) might be due to a background mutation. Indeed. This could be in the strain carrying the puf-8 (ok302) allele as they suggest. Alternatively, this could be a mutation that weakens the phenotype associated with puf-8(q725). A Cas-9-generated null allele, showing either q725 or ok302 phenotypes would settle the point (not absolutely necessary for publication)

Minor Points
Page 3
…maybe tune down the sentence stating that 'We now have a comprehensive molecular understanding of the pathway required for the execution of the ……as well as….'

For instance, endogenous egl-1 single-copy transcriptional and translational reporters (or antibodies) have never been used…

Page 4

I think that Derry and Schumacher lab papers relating to egl-1 mRNA regulation by micro RNAs should be equally cited. Indeed, the mir35 microRNA mediated phenotypes relating to EGL-1 expression and apoptosis seem to be highly penetrant (in the germ line).

Page 13 very minor point. Maybe spell out what the ATFS protein is which is used as a precipitation control.

Page 22, 23 The Material section would benefit from a list of DNA constructs used.

Page 24 and 25 Agarose pads are 4.5% in page 4 and 2% in page 5, please double check.
Page 26. Please double-check the last paragraph. Are the exact same mRNA probes used as in the Ray 2008 and the Sherrard 2017 paper?

Please double check that remnant text relating to previous Figures 4 and 5 is not in discussion or material and methods (I have not carefully checked this)

<u>Reviewer 3</u>

Advance summary and potential significance to field

Same as the first review.

Comments for the author

The authors have performed two new experiments and included the results in the revised manuscript. One, ced-3 mRNA coimmunoprecipitates specifically with PUF-8 from worm lysates. Two, mutations of the six PUF-8-binding elements (PMEs) in the ced-3 3' UTR enhances embryonic lethality of a temperature-sensitive allele of ced-9(n1653) (13.9% to 45.5%), which is similar to the effect observed with puf-8(q725) [13.9% to 66.3%] (new Fig5D). These are important results. Overall, what emerges from the data presented are two distinct aspects of PUF-8's anti-apoptotic function:

1. Mutations in puf-8 or the mutations of the six PBEs in the ced-9 3' UTR enhance the embryonic lethality of a ts allele of ced-9 (n1653ts). The increased embryonic lethality observed in puf-8(q725); ced-9(n1653ts) is abolished by the ced-3(n717) loss-of-function mutation. Additionally, co-IP results show that PUF-8 and ced-3 mRNA are present in the same complex. These results show that PUF-8 negatively regulates apoptosis in the embryo largely via the PBEs of ced-3 3' UTR.

© 2023. Published by The Company of Biologists under the terms of the Creative Commons Attribution License (https://creativecommons.org/licenses/by/4.0/).

2. Mutations in puf-8 cause both precocious and ectopic deaths in specific cell lineages. In the mother cell of one of the cells that precociously die in puf-8(q725) embryos, in situ hybridization experiments reveal that the ced-3 and ced-4 mRNA levels are elevated in puf-8(q725) embryos when compared to the wildtype.

My concerns:

1. The results summarized in point 1 above are clear and provide compelling evidence that PUF-8 has an overall anti-apoptotic function that is mediated, at least partly, via the ced-3 3' UTR. However, except for the change in ced-3 and ced-4 mRNA levels, there is no evidence that the anti-apoptotic effect of PUF-8 observed in the specific cells that precociously or ectopically die (results summarized in point 2 above) is mediated by its direct interaction with the ced-3 3' UTR in those cells. The lines 4-6 in the Abstract ("we present evidence that this anti-apoptotic activity of PUF-8 is dependent on PUF-8's ability to interact with ced-3caspase mRNA thereby repressing the activity of the pro-apoptotic ced-3caspase gene") are not supported by evidence. If the authors want to establish that PUF-8 prevents cell death in these specific cells by suppressing ced-3 posttranscriptionally by direct binding to the PBEs in the ced-3 3' UTR, then additional experiments to address the following are required:

a. Is there a PBE-dependent change in CED-3 protein level in any of the specific cells, for example in RIDnb?
b. Is there an upregulation of CED-3 protein in RIDnb upon depletion of PUF-8?

The above two questions are readily doable using reporter transgenes.

Importantly, the authors' new results that the PBE mutations in ced-3 3' UTR did not lead to precocious or ectopic cell deaths does not support that the anti-apoptotic activity of PUF-8 is via the ced-3 mRNA in the cells in which they have observed such cell deaths in the puf-8 mutant embryos.

2. The presence of ced-3 mRNA (detected by RT-PCR) in the IP pellet only shows that PUF-8 and ced-3 mRNA are present in the same complex; it does not reveal if PUF-8 directly binds to ced-3 mRNA. Thus, the conclusion in the Results subsection title on page 13 "PUF-8 protein interacts physically with cd-3 mRNA" is misleading. For this conclusion, an in vitro experiment such as EMSA is required. The authors use this phrase at multi places in the text, which should be revised to accurately reflect the results. Does the anti-FLAG antibody fail to co-IP the PBE-mutant ced-3 mRNA along with the FLAG-tagged PUF-8? This is certainly doable with the strains and reagents that the authors already have. Assaying the 3'UTR activity in a reporter transgene system [see Merritt et al. Current Biology 18, 1-7 (2008)] with wildtype and PBE mutant ced-3 3' UTR, in the wildtype and puf-8 mutant background, will unequivocally convince the regulatory relationship between an PUF-8 and the ced-3 mRNA.

3. The embryonic lethality for puf-8(q725); ced-9(n1653ts) is reported as 78.2 in Fig1E and 66.3% in Fig5D. I understand these variations are common in biology, but since these two are identical experiments with statistical analyses, it will be good to record this difference and explain the plausible reason(s) in a couple of lines so that the reader is not confused.

Second revision

<u>Author response to reviewers' comments</u>

Reviewer 1

Reviewer 1 Advance summary and potential significance to field

This manuscript demonstrates a direct interaction of the PUF-8 protein with the 3'UTR of the ced-3 gene, which regulates developmentally controlled apoptosis in C. elegans. This is an important advance because it describes a new and important layer of regulation of programmed cell death. This sets the stage for other groups to investigate conserved roles of homologous RNA-binding proteins in vertebrates.

© 2023. Published by The Company of Biologists under the terms of the Creative Commons Attribution License (https://creativecommons.org/licenses/by/4.0/).

Reviewer 1 Comments for the author

I am satisfied with revised manuscript and recommend publication.

Reviewer 2

Reviewer 2 Advance summary and potential significance to field
see below

Reviewer 2 Comments for the author
The revised manuscript by Xu et al., is very much improved and to my mind warrants publication with minor revisions. I think that it was wise to remove Figures 4 and 5 focused on apoptosis protein expression as such measurements in subcompartments of single cells and organelles (mitochondria CED-9) in the context if an entire organism are most difficult/ next to impossible, especially given that differentials are not expected to be 'black and white'. The new evidence showing directed PUF-8 ced-3 mRNA binding, and the binding sites being required in vivo are all the more convincing. Congratulations!

Major Point

Sorry to have missed this earlier on: It is imperative to provide a full strain list using 'Conradt lab' designations. Providing this is most important for other labs to use the reagents and, if necessary, reproduce the data. Most of the alleles used in this study were still generated by classic genetic, whole genome mutagenesis-based methods where modifying mutations may remain, even after excessive backcrossing. Indeed, the authors imply that the differences observed between the strains carrying puf-8(q725) and puf-8 (ok302) alleles (both of which are likely to be null alleles) might be due to a background mutation. Indeed. This could be in the strain carrying the puf-8 (ok302) allele as they suggest. Alternatively, this could be a mutation that weakens the phenotype associated with puf-8(q725). A Cas-9-generated null allele, showing either q725 or ok302 phenotypes would settle the point (not absolutely necessary for publication).

BC: We now provide a Supplementary Table (Table S1) which lists all strains used throughout this study including their complete genotype (Conradt lab strains as well as strains from other labs used). Concerning a Cas-9-generated null allele of puf-8, we decided not to generate such an allele because we consider it beyond the scope of this study.

Minor Points

Page 3
…maybe tune down the sentence stating that 'We now have a comprehensive molecular understanding of the pathway required for the execution of the ……as well as….'

BC: We have changed the sentence and now state:

"As a result of numerous studies, we have a molecular understanding of the pathway required for the execution of the cell death fate during C. elegans development, as well as the mechanisms through which the activity of this pathway is controlled (Horvitz 2003; Lettre and Hengartner 2006; Conradt et al. 2016)."

For instance, endogenous egl-1 single-copy transcriptional and translational reporters (or antibodies) have never been used…

Page 4

I think that Derry and Schumacher lab papers relating to egl-1 mRNA regulation by micro RNAs should be equally cited. Indeed, the mir35 microRNA mediated phenotypes relating to EGL-1 expression and apoptosis seem to be highly penetrant (in the germ line).

© 2023. Published by The Company of Biologists under the terms of the Creative Commons Attribution License (https://creativecommons.org/licenses/by/4.0/).

BC: Since the Derry and Schumacher papers were published 2 years after we initially described microRNA-mediated control of egl-1 expression and since their work exclusively focuses on the germline (rather than the soma), we prefer not to cite these two papers on page 4.

Page 13 very minor point. Maybe spell out what the ATFS protein is which is used as a precipitation control.

BC: We have added the following information on page 13 "(ATFS-1, Activating Transcription Factor Associated with stress-1). (Haynes et al, 2010)"

Page 22, 23 The Material section would benefit from a list of DNA constructs used.

BC: As suggested by the reviewer, we have added another Supplementary Table (Table S3), which lists the DNA constructs used throughout the study.

Page 24 and 25 Agarose pads are 4.5% in page 4 and 2% in page 5, please double check.

BC: We have double checked this and found that all agarose pads used in this study were 2%. This has been corrected in the manuscript.

Page 26. Please double-check the last paragraph. Are the exact same mRNA probes used as in the Ray 2008 and the Sherrard 2017 paper?

BC: Raj et al. 2008 established the method for smRNA FISH in mammalian cell lines, flies and nematodes. Sherrard et al. 2017 used the same probes for detecting egl-1 mRNA. We are now providing tables with the sequences of all probes used for the smRNA FISH experiments in this study (Table S4-S8).

Please double check that remnant text relating to previous Figures 4 and 5 is not in discussion or material and methods (I have not carefully checked this)

BC: We checked and did not find any references to the previous Figures 4 and 5.

Reviewer 3

Reviewer 3 Advance summary and potential significance to field

Same as the first review.

Reviewer 3 Comments for the author

The authors have performed two new experiments and included the results in the revised manuscript. One, ced-3 mRNA coimmunoprecipitates specifically with PUF-8 from worm lysates. Two, mutations of the six PUF-8-binding elements (PMEs) in the ced-3 3' UTR enhances embryonic lethality of a temperature-sensitive allele of ced-9(n1653) (13.9% to 45.5%), which is similar to the effect observed with puf-8(q725) [13.9% to 66.3%] (new Fig5D). These are important results. Overall, what emerges from the data presented are two distinct aspects of PUF-8's anti-apoptotic function:

1.Mutations in puf-8 or the mutations of the six PBEs in the ced-9 3' UTR enhance the embryonic lethality of a ts allele of ced-9 (n1653ts). The increased embryonic lethality observed in puf-8(q725); ced-9(n1653ts) is abolished by the ced-3(n717) loss-of-function mutation. Additionally, co-IP results show that PUF-8 and ced-3 mRNA are present in the same complex. These results show that PUF-8 negatively regulates apoptosis in the embryo largely via the PBEs of ced-3 3' UTR.

2.Mutations in puf-8 cause both precocious and ectopic deaths in specific cell lineages. In the mother cell of one of the cells that precociously die in puf-8(q725) embryos, in situ hybridization experiments reveal that the ced-3 and ced-4 mRNA levels are elevated in puf-8(q725) embryos when compared to the wildtype.

© 2023. Published by The Company of Biologists under the terms of the Creative Commons Attribution License (https://creativecommons.org/licenses/by/4.0/).

My concerns:

1.The results summarized in point 1 above are clear and provide compelling evidence that PUF-8 has an overall anti-apoptotic function that is mediated, at least partly, via the ced-3 3' UTR. However, except for the change in ced-3 and ced-4 mRNA levels, there is no evidence that the anti-apoptotic effect of PUF-8 observed in the specific cells that precociously or ectopically die (results summarized in point 2 above) is mediated by its direct interaction with the ced-3 3' UTR in those cells. The lines 4-6 in the Abstract ("we present evidence that this anti-apoptotic activity of PUF-8 is dependent on PUF-8's ability to interact with ced-3caspase mRNA thereby repressing the activity of the pro-apoptotic ced-3caspase gene") are not supported by evidence.

BC: Concerning lines 4-6 of the abstract, our finding that the loss of puf-8 does not increase embryonic lethality in ced-9(n1653ts); ced-3(bc448) animals indicates that mutating the PBEs in the ced-3 3'UTR prevents any effect that the loss of puf-8 has on embryonic lethality. This indicates that in a ced-9(n1653ts) background, puf-8 acts through the PBEs in the ced-3 3'UTR to block apoptosis (anti-apoptotic function). For this reason, we disagree with the reviewer and prefer not to change lines 4-6 in the abstract.

If the authors want to establish that PUF-8 prevents cell death in these specific cells by suppressing ced-3 posttranscriptionally by direct binding to the PBEs in the ced-3 3' UTR, then additional experiments to address the following are required:

a.Is there a PBE-dependent change in CED-3 protein level in any of the specific cells, for example in RIDnb?

b.Is there an upregulation of CED-3 protein in RIDnb upon depletion of PUF-8?

The above two questions are readily doable using reporter transgenes.

BC: In the previous manuscript, we presented experiments with which we had attempted to look at CED-3 protein levels in the RIDnb in puf-8 mutants compared to wild-type. However, this turned out to be very difficult. In response to the previous reviewers' comments, we decided to remove the figures describing these experiments. While we agree with the reviewer that the suggested experiments (a, b) could strengthen our model, we consider it currently technically not feasible.

Importantly, the authors' new results that the PBE mutations in ced-3 3' UTR did not lead to precocious or ectopic cell deaths does not support that the anti-apoptotic activity of PUF-8 is via the ced-3 mRNA in the cells in which they have observed such cell deaths in the puf-8 mutant embryos.

BC: As stated in 'Results' and 'Discussion', we propose that the anti-apoptotic function of PUF-8 protein is mediated by PUF-8's ability to repress expression of ced-3 AND ced-4 in cells programmed to survive. This is based on the observation that (in contrast to the loss of puf-8) ced-3(bc448) (PBE mutations) by itself does not cause embryonic lethality or the appearance of large cell corpses (ectopic/precocious cell death). In ced-3(bc448) mutants, the ability of PUF-8 to repress ced-3 expression is abolished but not PUF-8's ability to repress ced-4 expression.

2.The presence of ced-3 mRNA (detected by RT-PCR) in the IP pellet only shows that PUF-8 and ced-3 mRNA are present in the same complex; it does not reveal if PUF-8 directly binds to ced-3 mRNA. Thus, the conclusion in the Results subsection title on page 13 "PUF-8 protein interacts physically with ced-3 mRNA" is misleading.

BC: We agree with the reviewer that our IP indicates that ced-3 mRNA and PUF-8 protein are in the 'same complex', which we refer to as a 'physical interaction'. We do not claim that our IP results show that ced-3 mRNA and PUF-8 'directly' interact. Indeed, the conclusion of the Results subsection "PUF-8 protein interacts physically with ced-3 mRNA" on page 13 mentioned by the reviewer is:

© 2023. Published by The Company of Biologists under the terms of the Creative Commons Attribution License (https://creativecommons.org/licenses/by/4.0/).

"Based on these findings, we conclude that PUF-8PUM1, 2 protein interacts physically with ced-3caspase mRNA in vivo, and that it may also interact physically with ced-9Bcl-2 and ced-4Apaf-1 mRNAs."

For this reason, we see no reason to make changes.

For this conclusion, an in vitro experiment such as EMSA is required. The authors use this phrase at multi places in the text, which should be revised to accurately reflect the results. Does the anti-FLAG antibody fail to co-IP the PBE-mutant ced-3 mRNA along with the FLAG-tagged PUF-8? This is certainly doable with the strains and reagents that the authors already have. Assaying the 3'UTR activity in a reporter transgene system [see Merritt et al. Current Biology 18, 1-7 (2008)] with wildtype and PBE mutant ced-3 3' UTR, in the wildtype and puf-8 mutant background, will unequivocally convince the regulatory relationship between an PUF-8 and the ced-3 mRNA.

BC: We agree with the reviewer that these experiments would be informative; however, they are beyond the scope of the current study.

3.The embryonic lethality for puf-8(q725); ced-9(n1653ts) is reported as 78.2 in Fig1E and 66.3% in Fig5D. I understand these variations are common in biology, but since these two are identical experiments with statistical analyses, it will be good to record this difference and explain the plausible reason(s) in a couple of lines so that the reader is not confused.

BC: The two experiments were done by different individuals (Jimei Xu and Yanwen Jiang) in different labs (Conradt lab at LMU Munich, before 2020 and Conradt lab at UCL, after 2020) using different incubators. We have added the following statement to the legend of Figure 5:

"(Of note, percent embryonic lethality observed for puf-8(q725); ced-9(n1653ts) in the experiment presented in Fig. 1E is 78.2%. In the experiment presented in this figure it is 66.3%. The two sets of experiments were performed by two different individuals in two different laboratories.)"

Third decision letter

MS ID#: DEVELOP/2022/201167

MS TITLE: The C. elegans PUM1, 2-like RNA binding protein PUF-8 is required for robustness of the cell death fate

AUTHORS: Jimei Xu, Yanwen Jiang, Ryan Sherrard, Kyoko Ikegami, and Barbara Conradt
ARTICLE TYPE: Research Article

I am happy to tell you that your manuscript has been accepted for publication in Development, pending our standard ethics checks.

© 2023. Published by The Company of Biologists under the terms of the Creative Commons Attribution License (https://creativecommons.org/licenses/by/4.0/).
